# Dissecting FAP^+^ Cell Diversity in Pancreatic Cancer Uncovers an Interferon-Response Subtype of Cancer-Associated Fibroblasts with Tumor-Restraining Properties

**DOI:** 10.1158/0008-5472.CAN-23-3252

**Published:** 2025-04-11

**Authors:** Joshua Cumming, Parniyan Maneshi, Mitesh Dongre, Tala Alsaed, Mohammad Javad Dehghan-Nayeri, Agnes Ling, Kristian Pietras, Cedric Patthey, Daniel Öhlund

**Affiliations:** 1 Department of Diagnostics and Intervention, Umeå University, Umeå, Sweden.; 2 Wallenberg Centre of Molecular Medicine, Umeå University, Umeå, Sweden.; 3 Department of Medical Biosciences, Umeå University, Umeå, Sweden.; 4 Division of Translational Cancer Research, Department of Laboratory Medicine, Lund University, Lund, Sweden.

## Abstract

Within the stroma of pancreatic ductal adenocarcinoma (PDAC), mesenchymal cells differentiate into cancer-associated fibroblast (CAF) subtypes that differentially mediate disease progression. Defining the regulatory mechanism and diversity of CAF subtypes could identify potential therapeutic strategies to harness the tumor-suppressive activities of CAFs. To address this, we utilized single-cell RNA sequencing to profile fibroblast activation protein-α (FAP)–expressing mesenchymal cells in human PDAC. The mesenchymal subpopulations in PDAC reflected mesenchymal cell heterogeneity found in the normal developing pancreas. In addition to characterizing inflammatory CAF and myofibroblastic CAF subpopulations in detail, the analysis uncovered a previously undescribed interferon-response CAF (ifCAF) subtype. Tumor-derived signals induced specific CAF subtypes from pancreatic stellate cells in an organoid-based coculture model, and time-course experiments revealed regulatory mechanisms that govern subtype formation. STING agonists promoted an ifCAF phenotype *in vivo* and *in vitro*. Importantly, induction of an ifCAF phenotype suppressed tumor cell invasiveness and induced an antitumor phenotype in tumor-associated neutrophils. Together, this study resolves FAP^+^ stromal cell heterogeneity in PDAC and identifies an ifCAF subtype that can be induced to suppress protumorigenic features of PDAC.

**Significance:**

Characterization of FAP^+^ mesenchymal cell heterogeneity in pancreatic cancer identifies a tumor-suppressive interferon-response cancer-associated fibroblast subtype that can be induced by stimulating type I interferon signaling using STING agonists.

## Graphical Abstract

**Figure:**
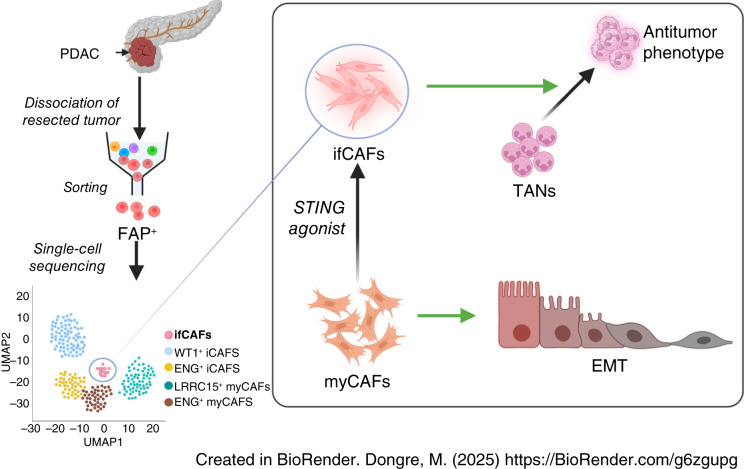

## Introduction

Pancreatic ductal adenocarcinoma (PDAC) is characterized by a desmoplastic stroma. Cancer-associated fibroblasts (CAF) dominate this tumor microenvironment (TME) in which they exert biological functions that shape the tumor architecture and regulate disease growth and spread (1).

CAFs in PDAC can arise from pancreatic stellate cells (PSC; ref. 2). PSCs are resident cells of the pancreas with characteristic cytoplasmic lipid droplets and expression of fibroblast activation protein-α (FAP; ref. 3). Upon receiving signals derived from cancer cells, PSCs can differentiate from a quiescent state into CAFs. Once activated, CAFs modulate tumorigenesis through secretion of fibrotic extracellular matrix (ECM) components and proinflammatory molecules (2, 4–7). Such roles may be tumor-promoting, with CAFs shaping the TME to facilitate immune suppression, therapy resistance, and tumor cell proliferation and metastatic spread (8–10). Through therapeutically targeting CAFs, and CAF-derived stromal components, these protumorigenic functions can be ablated, yielding improvements in drug delivery and immunotherapy (11–13). Contrarily, depleting CAFs through genetic or pharmacologic intervention has augmented tumor growth and given rise to invasive undifferentiated tumors in PDAC, which also indicates a tumor-restraining role for CAFs (14–16).

Cellular heterogeneity is a proposed mechanism through which CAFs can orchestrate divergent pathophysiologic functions. Distinct subpopulations of CAFs have been identified in PDAC (4, 17–19). We have previously identified inflammatory CAFs (iCAF) and myofibroblastic CAFs (myCAF; ref. 4). These subtypes are spatially and phenotypically distinct and can be selectively targeted to manipulate tumor growth (4, 7). In addition, antigen-presenting CAFs (apCAF) have been characterized and proposed to negatively regulate immune cell activity within the TME (17).

In this study, we use single-cell RNA sequencing (scRNA-seq) to dissect the heterogeneity of FAP^+^ cells in the TME of human tumors and demonstrate that a murine three-dimensional *in vitro* coculture model of PDAC can recapitulate CAF differentiation observed *in vivo*. Furthermore, we map pathways and transcription factors important in the induction and maintenance of different CAF subtypes and identify a novel interferon-response CAF (ifCAF) subtype in PDAC. This subtype is characterized by a type I interferon response and, in contrast to apCAFs, does not exhibit MHC class II antigen presentation. Through experiments in which ifCAFs are induced *in vivo* and *in vitro*, we show that ifCAFs exhibit antitumor activities by promoting a less invasive phenotype in tumor cells and by directly modulating the polarization of tumor-associated neutrophils (TAN). These findings are important to fully understand the diversity of CAF subtypes and for the future development of therapeutic interventions targeting CAFs.

## Materials and Methods

### Human tumor samples

Human PDAC tumor samples were resected at Umeå University Hospital. The study was conducted according to the Declaration of Helsinki and was approved by the ethical review board at Umeå University (09–175M) and the Swedish Ethical Review Authority (2019–00399). All subjects taking part in the study provided written informed consent. Samples were included when confirmed as PDAC through assessment by a pathologist. Six tumor samples from five patients were profiled.

### Mice

C57BL/*6J* mice (Jackson Laboratory, 000664, RRID: IMSR_JAX:000664) were housed in Umeå University’s Umeå Center for Comparative Biology facility. Ethical approval was acquired from the Swedish Board of Agriculture (A23–2022), and animals were housed according to Swedish rules on research.

### Cell line isolation and culture conditions

The PSC lines used had been isolated and immortalized as previously described (4). Murine pancreatic tumor organoids (mT) used were isolated from KrasLSL-G12D/+; Trp53LSL-R172H/+; Pdx-1-Cre (KPC) mice and cultured as previously described (4, 20).

PSCs were cultured on tissue culture–treated plastic in media composed of DMEM (Sigma-Aldrich) with 5% FBS (Gibco) and penicillin/streptomycin (Gibco, 15140122). PSCs were passaged using TrypLE Express (Gibco, 12605) to dissociate for 2 minutes at 37°C, washed in DMEM supplemented with 5% FBS, centrifuged, and resuspended in culture media before replating at a 1:20 ratio.

Organoids were cultured in Matrigel domes (Corning, 356231) with murine pancreatic organoid feeding media (21): Advanced DMEM/F12 (Gibco, 12634) supplemented with 1× GlutaMAX (Gibco, 35050), 1x HEPES (Gibco, 15630), 1× B27 (Invitrogen, 17504044), 1.25 mmol/L N-acetylcysteine (Sigma-Aldrich, A9165), 10 nmol/L gastrin (Tocris, 3006), 50 ng/mL EGF (Thermo Fisher Scientific, PMG8041), 10% RSPO1-conditioned media, 100 ng/mL Noggin (PeproTech, 250-38), 100 ng/mL FGF10 (PeproTech, 100-26), and 10 mmol/L nicotinamide (Sigma-Aldrich, N0636). To passage organoids, Matrigel was washed away using cold splitting media [Advanced DMEM/F12 supplemented with 1× GlutaMAX (Gibco) and 1× HEPES (Gibco)], and organoids were mechanically fragmented using small aperture glass pipettes and then seeded into fresh Matrigel at roughly a 1:5 ratio. Passaging was performed twice per week. All conditions for cocultures are described below following the suggested guidelines for coculture reporting (22).

### Transfection of cell lines with fluorophores

PSC-mCherry and mT-GFP fluorescent cell lines were generated as previously described (4).

For the PSC-mKate2 and mT-tGFP lines, lentiviral particles were generated by transfecting pLVX-EF1α-mKate2-WPRE-Neo (VectorBuilder) and pLVX-EF1α-turboGFP-WPRE-Neo (VectorBuilder) plasmids, respectively, into 293T cells (RRID: CVCL_0063). The target plasmids were transfected together with psPAX2 (Addgene, 12260, RRID: Addgene_12260) and pMD2.G (Addgene, 12259, RRID: Addgene_12259) packaging plasmids using Lipofectamine 2000 transfection reagent (Invitrogen, 11668027). Media was changed 24 hours later, and the lentivirus supernatant was collected after an additional 24 hours. The supernatant was filtered through a 0.45-µm filter and concentrated with Lenti-x concentrator (Takara Bio Inc., 631231) and 250 µL aliquots stored at −80°C.

To obtain turboGFP-labeled organoids, an organoid culture from a single well of a 24-well plate was mechanically dissociated into small fragments using a narrow aperture glass pipette and then digested briefly for 5 minutes at 37°C in TrypLE Express (Gibco), which was then quenched using DMEM (Sigma-Aldrich) with 5% FBS (Gibco), centrifuged and resuspended in a 250 µL aliquot of the pLVX-EF1α-turboGFP-WPRE-Neo lentivirus with 10 µg/mL polybrene infection/transfection reagent (Millipore-Sigma, TR-1003), transferred into a single well of a low-attachment 48-well plate and centrifuged for 1 hour at room temperature, and then incubated at 37°C 5% CO_2_ for 6 hours. The cell suspension was then collected in splitting media, centrifuged, and then resuspended and seeded in Matrigel with organoid media supplemented with 10.5 µmol/L ROCK inhibitor Y-27632 (Sigma-Aldrich) for the first passage. Two days later, media were exchanged with organoid media supplemented with 1 mg/mL G418 (Roche, G418-RO) to begin selection.

To obtain mKate2-labeled PSCs, pLVX-EF1α-mKate2-WPRE-Neo virus supplemented with 10 µg/mL polybrene infection/transfection reagent (Sigma-Aldrich) was incubated with PSCs grown in monolayer for 24 hours. Two days after infection, cells were treated with 1mg/mL G418 (Roche) for selection. Both the organoid and PSC cell lines were later enriched for highly fluorescent cells by FACS.

### Proliferation assays for monocultures and cocultures with gemcitabine and DMXAA

For proliferation assays, organoids and PSC were enzymatically dissociated to a singe-cell suspension. For cocultures, 1,000 mT-tGFP organoid cells and 2,000 PSCs were seeded in a volume of 24 µL of a 1:1 mixture of splitting medium and Matrigel in a well of a clear-bottom 384-well plate, resulting in the organoids being lined up at the same horizontal level. For organoid monoculture, 1,000 cells were seeded in the same conditions. Fifty-six µL of splitting medium was added, and the cells were grown at 37°C. After 24 hours, DMXAA and/or gemcitabine were added to a final concentration of 100 µmol/L or 5 nmol/L, respectively. After an additional 48 hours, the cultures were supplemented with 30 µL of splitting medium containing the same final concentration of drug. Control wells were treated with an equal amount of vehicle (DMSO for DMXAA and water for gemcitabine). The mT-tGFP fluorescence was measured in a single microsopy image every 24 hours for 6 days from the time of DMXAA/gemcitabine addition using a SpectraMax I3X with the green (541 nm, 10 milliseconds exposure) fluorescent channel. Images collected by the 828 Minimax imaging procedure were analyzed by the associated SoftMax Pro Data Acquisition and Analysis 829 Software (RRID: SCR_014240) version 7.0 (Molecular Devices). GFP fluorescence was quantified as covered area in the green channel.

### Orthotopic transplantation of tumor cells

Orthotopic transplantation was performed essentially as described previously (23). All surgical procedures were performed under stringent sterile practices. Briefly, murine tumor organoids were subcultured to 80% confluency and dissociated into single cells. Cells were resuspended in 30% Matrigel in the splitting medium at a concentration of 3.3 × 10^7^ cells/mL and stored on an ice bath. For transplantation, 5-week-old C57BL6/J mice were sedated with isoflurane, and a small incision was made in the left lateral abdomen to expose the pancreas and spleen. 1 × 10^6^ cells were injected directly into the splenic lobe (tail) of the pancreas before closing the incision with sutures and a skin clip. Successful transplants fulfilled three main criteria: a clear injection bubble is formed, no excessive leakage of cells into the peritoneal cavity, and no excessive postoperative bleeding. Postoperative care of the animals was done according to ethical guidelines. Tumor growth was monitored periodically by palpating the abdomen and by ultrasound. Mice with tumor diameter ∼8 to 10 mm (8–12 weeks after transplantation) were recruited randomly in the MSA-2 arm or the vehicle control arm of the study. The drug regime included oral gavage of 80 mg/kg MSA-2 (MedChemExpress, HY-136927) or vehicle control (50% PEG300 (Sigma-Aldrich, 202371) in 0.9% NaCl) on days 0 and 5. On day 6, mice were euthanized by CO_2_, and tumors were harvested and processed for scRNA-seq profiling.

### Dissociation, staining, and FACS of human PDAC and mouse samples

Tumor specimens were thoroughly minced in mincing media [DMEM (Sigma-Aldrich, D5796) with 10% FBS (Gibco, 12657029) and DNase I (0.2 mg/mL, Sigma-Aldrich, D5025)]. Tumor fragments were collected and pelleted at 400 Relative Centrifugal Force (RCF) for 5 minutes at room temperature. The supernatant was aspirated, and the tumor pellet was resuspended in 5 mL digestion media [DMEM with 10% FBS, DNase I (0.2 mg/mL), and collagenase D (2.5 mg/mL, Sigma-Aldrich, 11088882001) and Liberase DL (0.5 mg/mL, Sigma-Aldrich, 5466202001)] and digested at 37°C for 45 minutes with gentle agitation. The reaction was quenched with 1 mL FBS and 500 µL defined trypsin inhibitor (DTI, Thermo Fisher Scientific, R007100; human) or 5 mL mincing media and 2 mL DTI (mouse). The digested fragments were further mechanically dissociated through triturating 20 to 30 times with a 5-mL pipette before passing through a 100-μm cell strainer (Thermo Fisher Scientific, 15380801) to yield a cell suspension. Cells were centrifuged (all subsequent centrifugations 400 RCF 5 minutes at 4°C) and resuspended in 3 mL ACK lysis buffer (Gibco, A1049201) and left on ice for 3 minutes before quenching with flow buffer (FB; human: 2% FBS in PBS; mouse: 2% FBS and 2.5% DTI in PBS). Cells were centrifuged and re-suspended in staining buffer (SB; human: 0.5% BSA and 2 mmol/L EDTA in PBS; mouse: 0.5% BSA, 10% DTI, and 2 mmol/L EDTA in PBS) containing either human FC receptor-binding inhibitor (Thermo Fisher Scientific, 14-9161-73, RRID: AB_468582, 1:20) or purified anti-mouse CD16/32 (BioLegend, 101302, RRID: AB_312801, 1:25) on ice for 15 minutes. For human tumors, excess SB was added, and cells were pelleted before resuspension in SB containing anti–CD45-FITC (Thermo Fisher Scientific, 11-0459-42, RRID: AB_10852703, 1:20), anti–EPCAM-A647 (BioLegend, 324212, RRID: AB_756086, 1:20), anti–CD31-SB600 (Thermo Fisher Scientific, 63-0319-42, RRID: AB_2717033, 1:20), and anti–FAP-PE (R&D Systems, FAB3715P-100, 1:40) or mouse IgG1-PE control (R&D Systems, IC002P, RRID: AB_357242, 1:40). For mouse tumors, excess SB was added, and cells were pelleted before resuspension in SB containing anti–CD45-APC (Thermo Fisher Scientific, 17-0451-82, RRID: AB_469392, 1:100), anti–EpCAM-FITC (Thermo Fisher Scientific, 11-5791-82, RRID: AB_11151709, 1:200), and anti–PDPN-PE (Thermo Fisher Scientific, 12-5381-82, RRID: AB_1907439, 1:100). Cells were spun down and resuspended in FB containing 4′,6-diamidino-2-phenylindole (DAPI; 1 μg/mL, Sigma-Aldrich, 10236276001) Sigma-Aldrich, 10236276001) on ice for 5 minutes. Cells were pelleted and resuspended in FB and passed through a 70-μm cell strainer (Thermo Fisher Scientific, 15346248). For human tumors ≥30,000 DAPI^−^/CD45^−^/CD31^−^/EPCAM^−^/FAP^+^ cells were sorted. For mouse tumors ≥30,000 DAPI^−^/CD45^+^, DAPI^−^/EpCAM^+^, DAPI^−^/PDPN^+^, and DAPI^−^/CD45^−^/Epcam^−^/PDPN^−^ cells were sorted. Cells were sorted into 20% FBS in PBS using a FACS Aria III cell sorter (BD Biosciences).

### Human and mouse PDAC sample single-cell capture, library preparation, and RNA-seq

For mouse tumors, equal numbers of sorted cell populations were mixed together. Cells were centrifuged and resuspended at 1,000 cells/mL in 0.2% BSA in PBS. A total of 12,800 to 15,000 cells were loaded per well into a 10× Chromium cartridge (10× Genomics, 1000127). cDNA and libraries were prepared across 6 samples derived from 5 human PDAC tumors or 11 cDNA and libraries across 6 samples derived from 6 mouse PDAC tumors according to the manufacturers’ instructions (Chromium Single Cell 3′ v3.1 protocol). cDNA libraries were sequenced on an Illumina NovaSeq 6000 S2-100 v1.5 flow cell at the Science for Life Laboratory National Genomic Infrastructure.

### Data processing of scRNA-seq profiling of human and mouse PDAC

**Figure 1.**
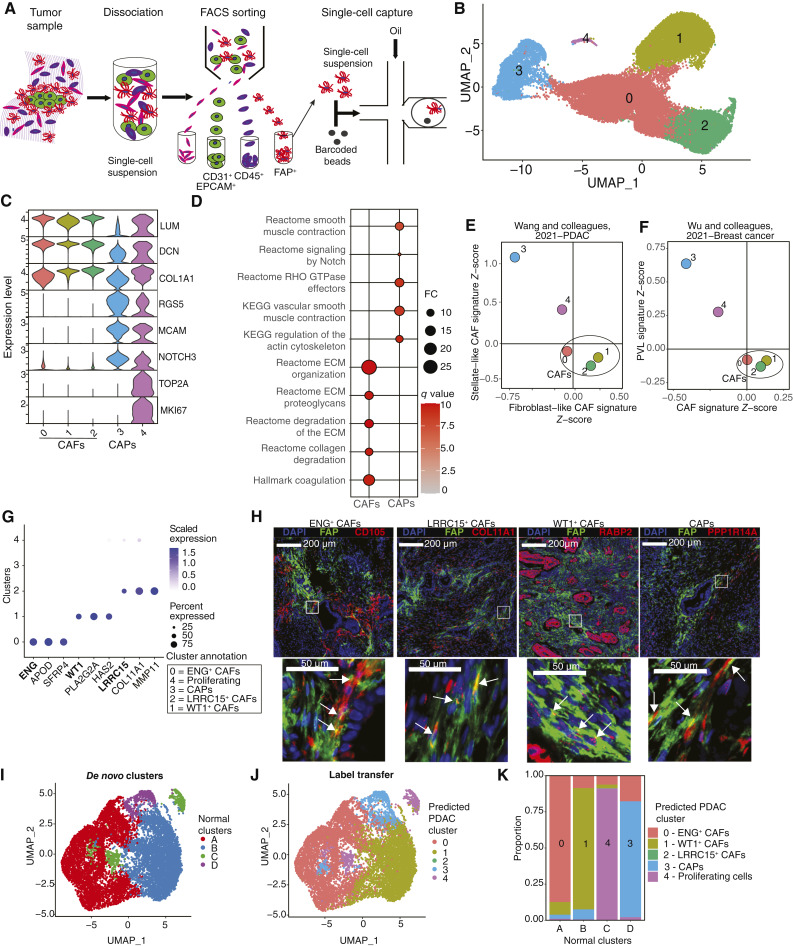
FAP^+^ mesenchymal subtypes populate the PDAC stroma and reflect mesenchymal heterogeneity of the healthy developing pancreas. **A,** Single-cell mRNA sequencing workflow depicting isolation and single-cell transcriptomics (droplet capture) of PDAC-derived DAPI^−^/CD45^−^/EPCAM^−^/CD31^−^/FAP^+^ cells. **B,** Unsupervised clustering of single cells for six cDNA libraries derived from five PDAC specimens visualized through UMAP. **C,** Stacked violin plots showing normalized gene expression levels of mesenchymal markers. **D,** GSEA showing enriched Hallmark, KEGG, and Reactome pathways in CAFs and CAPs. Selected pathways visualized in a dot plot. Size and color gradients represent FC and *q* values of enriched pathways, respectively. **E** and **F,** Scatter plots depicting mean subtype signature *Z*-scores from Wu and colleagues (**E**; ref. 19) and Elyada and colleagues (**F**; ref. 17). Each point represents a cluster as defined in **B**. **G,** Dot plot displaying DEG markers of CAF subtypes. The size of dots represents the percentage of cells in the cluster in which gene expression is detected, and the color represents scaled expression values. **H,** Representative immunofluorescence costaining of FAP and markers of CAF subpopulations (CD105, COL11A1, CRABP2, and PPP1R14A; red) in human PDAC (*n* = 5). Counterstain, DAPI (blue). Bottom, higher magnification illustrating costaining in cells (white arrows). The colors of markers are indicated above each image. **I,** Unsupervised clustering of mesenchymal cells derived from the normal developing pancreas from Olaniru and colleagues (27), visualized through UMAP. **J,** Same UMAP as in **I** colored by cell identity predicted through label transfer. **K,** Bar plots displaying proportion of predicted cell identity within *de novo* clusters. Colored by predicted cell identity.

**Figure 2.**
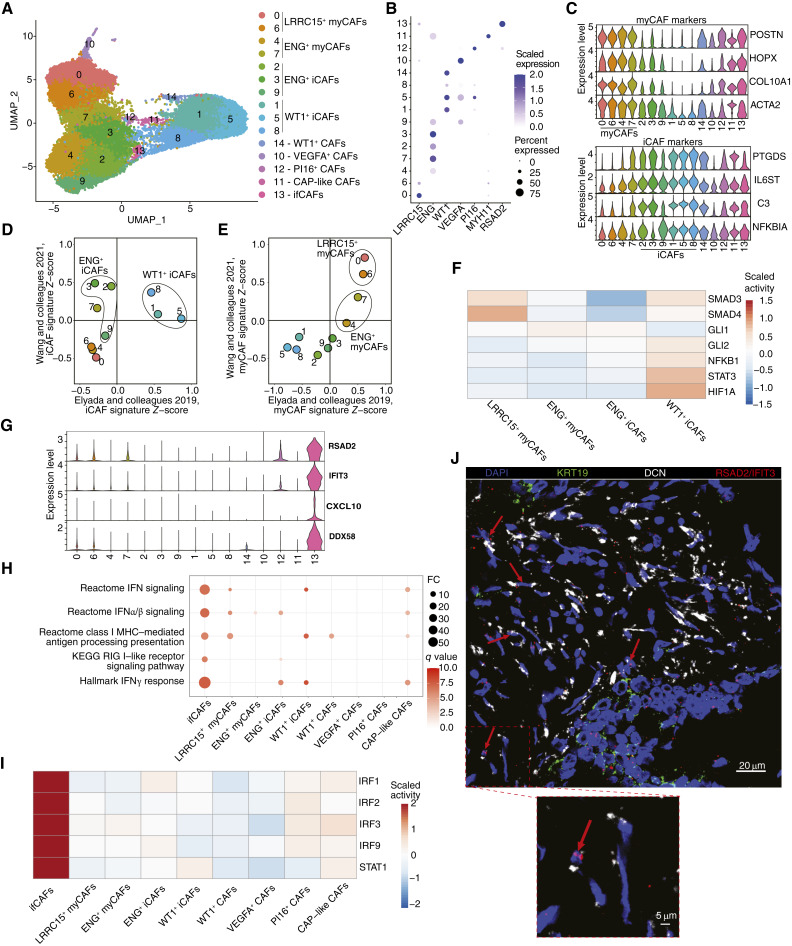
Characterization of FAP^+^ CAF subtypes in PDAC. **A,** UMAP displaying iterative unsupervised clustering of CAFs (clusters 0, 1, and 2 from Fig. 1B). **B,** Dot plot displaying DEG markers of CAF subtypes. The size of dots represents the cell percentage in the cluster in which gene expression is detected. Color quantifies expression values. **C,** Stacked violin plots showing normalized gene expression levels of myCAF (top) and iCAF (bottom) markers. **D** and **E,** Scatter plots depicting mean gene signature *Z*-scores for iCAFs (**D**) and myCAFs (**E**) from Wu and colleagues (19) and Elyada and colleagues (17) in clusters. Each point represents a cluster. **F,** Heatmap of scaled activity scores for selected transcription factor activities in iCAF and myCAF subtypes. **G,** Stacked violin plots showing normalized gene expression levels of ifCAF markers. **H,** GSEA showing enriched Hallmark, KEGG, and Reactome pathways in ifCAFs. Selected pathways visualized in a dot plot, in which the size and color of dots represent the FC in pathway enrichment and *q* values, respectively. **I,** Heatmap of scaled expression levels for differentially active transcription factors in ifCAFs. **J,** Top, representative image of FISH staining of FFPE sections of human PDAC. Red arrows highlight cells coexpressing *DCN* and *IFIT3/RSAD2*. Bottom, magnification of region of interest within the section (red square). The color of markers on each image are indicated.

FASTQ files were processed with the Cell Ranger pipeline version 6.1.2 with default parameters (10× Genomics, freeware, RRID: SCR_017344), mapping reads on the refdata-gex-mm10-2020-A reference genome. Raw read counts for each sample were first subjected to an initial round of quality control using the Seurat R package standard clustering pipeline with default parameters (Seurat_4.1.0, RRID: SCR_016341; ref. 24). The SoupX R package (SoupX_1.6.2, RRID: SCR_019193) was then used for estimation and removal of cell-free mRNA contamination using the standard pipeline (only in human samples; ref. 25). The “strained” read counts with ambient RNA contamination removed were then clustered using Seurat as previously described with the following amendment to default parameters: FindClusters used a resolution of 1.2. Clusters with markedly low read counts, low numbers of genes detected, and high percentages of mitochondrial genes were removed using the subset function. Subseted data were again clustered with Seurat as initially described. The DoubletFinder R package (DoubletFinder_2.0.3, RRID: SCR_018771) was next used to remove predicted doublets using default parameters (26). The number of expected doublets was defined using the initial number of cells before filtering and the expected doublet frequency from the Chromium Single Cell 3ʹ dual indexing v3.1 protocol. A final quality control step was performed to remove any remaining cells with a low number of genes detected and a high percentage of mitochondrial genes (nFeature_RNA > 1,000 and percent.mt < 10). Individual samples were then merged and processed in Seurat as described above with the following amendment: cell-cycle genes (Seurat cell-cycle scoring and regression vignette) expression were “regressed out” using the vars.to.regress feature of the ScaleData function. A total of 3,000 highly variable genes were identified for dimensionality reduction. The number of dimensions (principal components) for computing K-nearest neighbor graphs and shared nearest neighbor graphs and visualizing data with Uniform Manifold Approximation and Projection (UMAP) was defined as the last principal component at which the percentage change of variation is >0.1%. Individual samples were integrated using the reciprocal principal component analysis (rPCA) reduction method from Seurat with default parameters. For low-resolution clustering, a resolution of 0.1 was used, and for high-resolution clustering, a resolution of 0.6 was used. Differentially expressed genes (DEG) were called for using the FindMarkers function with min.pct = 0.25, fold change (FC) ≥ 1.5, adjusted *P* value ≤ 0.05 and only.pos = T. Label transfer was performed using the FindTransferAnchors function in Seurat with dimensions 1:10. The *Z*-score for each gene of a signature was defined as *Z* = (*x* − *μ*)/*σ*, in which *x* is the log-normalized unique molecular identifier (UMI) count, *μ* is the mean, and *σ* is the SD of the log-normalized UMI count for the gene, respectively. The mean *Z*-score of all genes in the gene signature was then taken to define a *Z*-score for a gene signature in an individual cell, and the mean of all cells in a cluster was taken as the cluster average *Z*-score (Figs. 1E, F, 2D and E; Supplementary Fig. S2D and S2E). Violin plots and feature plots for individual genes and gene signature *Z*-scores were generated in Seurat. Cluster trees were plotted using the clustree R package (clustree_0.4.4, RRID: SCR_016293) in combination with Seurat to perform clustering at indicated resolutions.

### Gene set enrichment analysis

For gene set enrichment analysis (GSEA) the R package SCPA (1.5.3) was used following the recommended workflow with default parameters (28). Hallmark, Kyoto Encyclopedia of Genes and Genomes (KEGG), and Reactome pathways were downloaded using the msigdbr R package (7.5.1, RRID: SCR_022870; ref. 29). Each cluster was compared against all other clusters, and significantly enriched pathways, FC >1 and adjusted *P* value < 0.05, were defined. Pathways were ranked on *q* values (*q*) and FC (*q* + *F*C), and pathways were manually selected from the top enriched pathways.

### Transcription factor activity inference

The gene regulatory networks were derived from the OmniPath database (30). The database was filtered for transcription factors with ≥20 target genes. The decoupleR R package (2.0.1) was subsequently used to estimate transcription factor activity using univariate linear modeling (31). The run_ulm function was used with modified parameters (minsize = 20) to infer activities. Activity scores per cell were centered using the ScaleData function in Seurat. Differentially active transcription factors were identified with the FindMarkers function on the scaled data. The pheatmap R package (1.0.12, RRID: SCR_016418) was used for plotting differentially active transcription factors in clusters.

### Cell-to-cell interaction analysis

The CellChat R package (RRID: SCR_021946; ref. 32) was used to infer communication weights between cell types following the recommended workflow with default parameters. The NicheNet R package (RRID: SCR_023158; ref. 33) was used to infer ligand target interactions following the recommended workflow with default parameters.

### Histopathology and immunofluorescence staining of human PDAC

Formalin-fixed paraffin-embedded tumor tissue blocks were acquired from the pathology biobank according to ethical permit number 2019–00399. All immunostainings were performed on 5-µm sections. Tumor areas were annotated, and the ECM was classified as dense, moderate, or loose by a pathologist. Hematoxylin and eosin staining was performed according to standard protocols. 2-Plex immunoperoxidase staining was performed with automated Discovery Ultra (Ventana Medical System, Inc.) according to the manufacturer’s instructions. After deparaffinization was completed at 69°C, antigen retrieval was performed for either 40 (for COL XI and CRABP2) or 64 (for CD105 and PPP1R14a) minutes with ultra cell conditioning solution 1 (Ultra CC1, pH 6, Ventana Medical Systems) at 95°C. To quench the endogenous peroxidase activity, slides were incubated with Discovery inhibitor (Ventana Medical Systems) for 8 minutes. Then, slides were incubated with the first primary antibodies (rabbit anti–COL XI (Proteintech, 21841-1-AP, RRID: AB_2918074, 1:400), rabbit anti-PPP1R14A (Thermo Fisher Scientific, PA5-83590, RRID: AB_2790743, 1:50), rabbit anti-CRABP2 (Abcam, ab181255, 1:100), and mouse anti-CD105 (Thermo Fisher Scientific, MA5-17041, RRID: AB_2538513) for 32 minutes at 36°C. Discovery OmniMap anti-rabbit HRP (Roche Diagnostics, 760-4311, RRID: AB_2811043) or Discovery OmniMap anti-mouse HRP (Roche Diagnostics, 760-4310, RRID: AB_2885182) secondary antibody was incubated on the slides for 16 minutes followed by the visualization of the immunoreaction with rhodamine and hydrogen peroxide for 16 minutes. Sections were then treated with Ultra Cell Conditioning Solution 2 (pH 6, Ventana Medical Systems) for 8 minutes at 100°C to denature the antibodies and the HRP (denaturing controls were performed and analyzed to ensure true staining). The second sequence of antibody staining was performed similarly to the first sequence with rabbit anti-FAPα (Abcam, ab240989, 1:100) and visualized with FITC. Nuclei were stained with DAPI for 8 minutes, and slides were washed and mounted using mounting medium (ibidi, 50001). Slides were scanned using Pannoramic 250 Flash III Digital Slide Scanner (3DHISTECH) and processed similarly with Slide Viewer (3DHISTECH).

### RNA *in situ* hybridization

*In situ* mRNA expression was assessed in formalin-fixed paraffin-embedded human PDAC sections from three separate patients. The RNAscope kit (Advanced Cell Diagnostics) was used according to the manufacturers’ instructions with 6-hour incubation with probes. Transcripts were detected using target probes for decorin (*DCN*), Keratin-19 (*KRT19*), interferon-induced protein with tetratricopeptide repeats 3 (*IFIT3*), and radical S-adenosyl methionine domain-containing 2 (*RSAD2*). Negative control probes (C1–C4) were subjected to identical treatment as target probes. *KRT19* and C3 negative control probes were detected with Opal 520 (1:750), *DCN* and C4 negative control probes were detected with Opal 690 (1:750), *IFIT3, RSAD2,* and C1 and C2 negative control probes were detected with Opal 570 (1:750). Slides were imaged using a Zeiss LSM 710 confocal microscope. Six or more optical sections were imaged for each section. Negative controls were used to set imaging parameters to detect positive staining. Images were analyzed using ZEN 3.3 (Blue Edition) software.

### Cell culture conditions for monocultures and cocultures for scRNA-seq profiling at day 6

For monocultures, 4 × 10^4^ PSCs were seeded in Matrigel domes and cultured for 6 days in DMEM (Sigma-Aldrich) containing 5% FBS (Gibco) and 1% penicillin/streptomycin (Gibco). Two PSC lines (PSC4-mCherry and PSC5-Cherry) were used in monoculture. For cocultures 1.5 × 10^4^ PSCs were seeded alongside murine tumor cells (mTs). mTs were taken from a confluent 3D culture, fragmented mechanically using a fire-polished pipette, and then seeded at a 1:4 ratio. Coculture of PSCs and mTs were performed as follows: PSC4-mCherry with mT4-GFP and PSC5-mCherry with mT3-GFP. Cocultures were cultured for 6 days in DMEM media containing 5% FBS and 1% penicillin/streptomycin.

### Cell culture conditions for monocultures and cocultures for scRNA-seq profiling of time course

For day 0 monocultures 5 × 10^4^ cells were seeded in Matrigel domes for 2 days in DMEM containing 5% FBS and 1% penicillin/streptomycin. To revert cells to a quiescent state prior to coculture with tumor cells, 1 × 10^5^ cells were seeded in Matrigel domes for 2 days. After 2 days, PSCs were dissociated to single cells from Matrigel domes as follows: Matrigel domes were gently dislodged using a pipette tip. Matrigel domes were then resuspended in Dispase (Thermo Fisher Scientific, 17105041) at 2 mg/mL in splitting media consisting of Advanced DMEM (Gibco) supplemented with 1× GlutaMAX (Gibco), 1× HEPES (Gibco), and 1% penicillin/streptomycin and digested at 37°C for 20 minutes. The reaction was quenched using splitting media, and cells were centrifuged at room temperature (all centrifugations at 400 RCF). Cells were resuspended in prewarmed TrypLE (Gibco) with 0.1 mg/mL DNase I (Sigma-Aldrich) and incubated for 5 minutes at 37°C. The reaction was quenched with DMEM 5% FBS and triturated ∼10×. Cells were spun and resuspended in splitting media and kept on ice. mTs were taken from a confluent 3D culture, fragmented mechanically using a fire-polished pipette, and then seeded at a 1:4 ratio with 3 × 10^4^ of the dissociated PSCs per well. Cocultures were cultured for 1 to 6 days in splitting media. PSC5-mKate2 and mT3-tGFP cell lines were used in the time-course experiments with two technical replicates at each time point.

### Dissociation and FACS sorting for scRNA-seq of monocultures and cocultures

Monocultures and cocultures were dissociated as described above. The single-cell suspension was spun down at 4°C and resuspended in 2% FBS in PBS with 1 µg/mL DAPI (Sigma-Aldrich) for 5 minutes, and cells were kept on ice from this point. Cells were spun down at 4°C and resuspended in 2% FBS in PBS before being passed through a 70 µm cell strainer into FACS tubes. tGFP^+^/mKate^−^ and tGFP^−^/mKate^+^ cells were sorted using BD FACSAria III into 20% FBS in PBS.

### Single-cell capture, library preparation, and RNA-seq of monocultures and cocultures

Cells collected from FACS were spun down, washed in 0.1% BSA (Sigma-Aldrich, A4503), pelleted, and then re-suspended at 3,500 cells/μL. A total of 14,000 cells were loaded in chambers of a ddSeq cartridge according to the standard protocol of SureCell Whole Transcriptome Analysis 3ʹ Library Prep Kit (6-cartridge kit; Illumina Bio-Rad SureCell, 20014280). Single-cells were captured using the ddSeq Single-Cell Isolator (Bio-Rad). Following single-cell capture, libraries were generated according to the manufacturer’s instructions for SureCell Whole Transcriptome Analysis 3ʹ Library Prep Kit. Single-cell libraries were then sequenced using NextSeq 500/550 Mid Output Kit v2.5 (150 cycles; Illumina, 20024904) and NextSeq 500/550 High Output Kit v2.5 (150 cycles; Illumina, 20024907).

### scRNA-seq data analysis of ddSeq-generated libraries

Illumina base call files were converted to FASTQ using the default Illumina bcl2fastq Conversion Software v2.20 (bcl2fastq, RRID: SCR_015058) pipeline with recommended parameters. FASTQ files from NextSeq 500/550 Mid Output Kit v2.5 and NextSeq 500/550 High Output Kit v2.5 were merged when libraries were sequenced on multiple flow cells. Custom scripts were used to generate the count matrix. Briefly, barcodes were deconvoluted by pattern matching of whitelisted barcodes allowing one mismatch. Nonmatched barcodes were further deconvoluted by alignment to the matched barcodes using CD-HIT (RRID: SCR_007105; ref. 34). After mapping to the GRCm38 mouse reference genome with STAR (RRID: SCR_004463; ref. 35), reads were given a gene annotation by assessing their overlap with exons of the Ensembl release 100 annotations using BEDTools (RRID: SCR_006646; ref. 36). UMIs were flattened, and the UMI count per gene and per cell was calculated. Cellular barcodes were selected with a knee plot method in which the barcode abundance cutoff was determined manually. The count matrix was imported into R for downstream analysis using Seurat as described above with the following amendments: The SoupX R package was not used as it is compatible only with the 10× Chromium sequencing platform. For scRNA-seq profiling, the time-course rPCA integration was not used. For scRNA-seq profiling of monocultures and cocultures on day 6, only rPCA was used to integrate technical and biological replicates (batches). Conversion between human and mouse genes for label transfer and *Z*-score calculations were performed using a human–mouse orthology table based on the Ensembl release number 98 orthology annotation available on BioMart (RRID: SCR_019214; Supplementary Table S7).

### Constructing single-cell trajectories and time-course analysis

The Slingshot (2.8.0, RRID: SCR_017012) R package was used to construct trajectories, define lineages, and estimate pseudotime from our scRNA-seq data using the recommended workflow (37). UMAP embeddings, cluster annotations, and read counts for highly variable genes were obtained from a Seurat object, generated as described above. To prevent cells from an early time point with a high pseudotime value from influencing the subsequent analysis, we used a pseudotime cutoff for cell selection to remove cells above this pseudotime in the lineage (indicated by dotted line in Fig. 4I).

To test for correlations between pseudotime values and transcription factor activities, generated as described above, for different lineages we used the Pearson product moment correlation coefficient and associated *P* values using the cor.test function in R.

To perform GSEA, genes were ranked and selected based on correlation between pseudotime values and normalized gene counts for each lineage. The Pearson product moment correlation coefficient and associated *P* value were used with the cor.test function in R. Only genes that were expressed in ≥10% of cells were retained. *P* values were adjusted to correct for multiple comparisons using the Bonferroni method. For each lineage, significantly correlated genes were selected as adjusted *P* value < 0.05 and Pearson correlation coefficient >0.25. Significantly correlated genes were ranked based on the Pearson correlation coefficient for GSEA using the R package clusterProfiler (4.2.2, RRID: SCR_016884; ref. 38). The recommended clusterProfiler workflow was used for subsequent analysis. To define KEGG pathways, we used the enrichplot (1.14.2; ref. 39) and DOSE R packages (40). For defining Reactome pathways that were enriched, we used the ReactomePA R package (RRID: SCR_019316; ref. 41). Hallmark pathways were downloaded using the msigdbr R package (7.5.1). Displayed pathways were manually selected among significantly enriched pathways (Supplementary Table S8) with adjusted *P* value ≤ 0.05.

### Cell culture, dissociation, and FACS sorting for bulk RNA-seq of PSC and tumor cell monocultures and cocultures

For monocultures, 5 × 10^4^ PSCs were seeded in Matrigel domes and cultured for either 4 or 6 days. Two PSC lines (PSC4-mKate2 and PSC5-mKate2) were used in monoculture. For cocultures, 2.5 × 10^4^ PSCs were seeded alongside mTs. mTs were taken from a confluent 3D culture, fragmented mechanically using a fire-polished pipette, and then seeded at a 1:4 ratio. Cocultures of PSCs and mTs were performed as follows: PSC4-mKate2 with mT4-tGFP and PSC5-mKate2 with mT3-tGFP. Cocultures were cultured for either 4 or 6 days. Cultures were established in splitting media or splitting media with recombinant murine IFNγ 10 ηg/mL ( R&D Systems, 485-MI-100/CF). After 4 days, media were refreshed with splitting media or splitting media with IFNγ. For STING agonist treatments, 100 µmol/L DMXAA (Tocris, 5601) or 0.5% DMSO (Sigma-Aldrich, D8418-100ML) were added in splitting media either for 6 hours on day 6 (Supplementary Fig. S5) or for 24 hours on day 3 (Fig. 6) before harvesting. For STING inhibitor treatment, 25 µmol/L H151 (Tocris, 6675) was added for 24 hours on day 3 before harvesting. T cells were isolated and added to cultures as follows. Spleens were isolated from C57BL/*6J* mice and finely minced in 2% FBS in PBS. Minced spleen was passed through 100-µm strainer and centrifuged. Cells were resuspended in ACK lysis buffer and left on ice for 3 minutes before reaction was quenched with 2% FBS in PBS. Cells were centrifuged and resuspended in 2% FBS in PBS. T cells were isolated using EasySep Mouse T Cell Isolation Kit (STEMCELL technologies, 19851) according to the manufacturer’s protocol. A measure of 1 × 10^5^ T-cells were added to the supernatant surrounding Matrigel domes in T-cell media [RPMI-1640 (Sigma-Aldrich, R8758-6X500ML), 10% FBS, 1.5% HEPES, 1% GlutaMAX, 0.5% penicillin/streptomycin, 0.2% β-mercaptoethanol (Thermo Fisher Scientific, 31350010)] or T-cell media alone. T cells were activated through addition of Dynabeads Mouse T-Activator CD3/CD28 for T-Cell Expansion and Activation (Thermo Fisher Scientific, 11456D).

After 6 days, cultures were dissociated to single cells as described above. For antibody staining, cells were blocked for nonspecific binding sites with anti-CD16/CD32 antibodies (BioLegend, 101302, RRID: AB_312801) for 20 minutes on ice in FB. Antibodies against MHC class II (Thermo Fisher Scientific, 62-5321-82, RRID: AB_2688070) were incubated for 30 minutes on ice in SB. Prior to sorting, cells were resuspended in either 2% FBS in PBS with 1× DAPI for 5 minutes or using LIVE/DEAD Fixable Near-IR Dead Cell Stain Kit (1:1,000 in PBS; Thermo Fisher Scientific, L34976) for 15 minutes and washed in 2% FBS in PBS before being passed through a 70 µm cell strainer into FACS tubes. A total of 500 cells were sorted using BD FACSAria III into 5 µL lysis buffer [dH2O, 0.1% Triton X-100 (Sigma-Aldrich, X100-500ML), and RiboLock RNase inhibitor (1 U/μL, Thermo Fisher Scientific, EO0381)]. Lysates were immediately frozen on dry ice and transferred to −80°C prior to library preparation.

### Neutrophil isolations from bone marrow, culture conditions, and bulk RNA-seq

C57Bl/6J mice were euthanized with CO2 based on the University guidelines. The mouse’s hind limbs and abdomen skin were disinfected using 70% ethanol. The abdominal cavity was opened with an incision on the midline of the lower abdomen and extended down the hind limbs past the ankle joint. The skin and adjoining quadriceps muscles were pulled back to expose the femur and the hip joint. Carefully holding the femur shaft, excess soft tissue and muscles on the head of the femur were removed. The pelvic-hip joint was cut to release the hind leg, and the tibia was cut off below the tibiofemoral joint without damaging the femur. Additional connective tissue and muscles were removed, and both ends of the femur were cut with a sharp scalpel. Femur and tibial bones from five C57B6 mice were washed in serum-free DMEM (SF-DMEM; DMEM, 1% 1 mol/L HEPES 1% Pen/Strep) on ice. Bone marrow was flushed from bones with SF-DMEM using a 21G syringe and pooled on ice. Cells were pelleted at 4°C and resuspended in ACK lysis buffer on ice for 3 minutes and then quenched with excess of SF-DMEM. 2 mL of cell solution added to a Percoll gradient (2 mL 78% Percoll in PBS, 2 mL 69% Percoll in PBS, and 2 mL 52% Percoll in PBS) at room temperature °C before centrifugation at 1,500 RCF at room temperature °C for 30 minutes. Neutrophils were collected between 72% and 69% layer using a Pasteur pipette in an excess of SF-DMEM. Cells were pelleted and resuspended in SB (0.5% BSA and 0.2 mg/mL DNAse-1 in SF-DMEM) with purified anti-mouse CD16/32 (1:20) and left on ice for 10 minutes. Cells were stained in anti–CD45-APC (1:100), anti–Ly6G-FITC (Thermo Fisher Scientific, 11-9668-82, RRID: AB_2572532, 1:200), and anti–CD11b-PE (Thermo Fisher Scientific, 12-0112-82, RRID: AB_2734869, 1:100) with DAPI (1 µg/mL) on ice for 30 minutes. Cells were washed in SB and passed through a 70-µm filter before ∼5 × 10^6^ neutrophils (CD45^+^/Ly6G^+^/CD11b^+^) were collected in SB. Neutrophils were then resuspended in SF-DMEM at 5,000 cell/μL.

For preparation of conditioned media (CM), 3 × 10^6^ PSCs were seeded in adherent 100-mm cell culture plates and cultured in SF-DMEM for 2 to 6 hours before addition of DMXAA to final concentration of 100 μmol/L or 0.2% DMSO. PSCs were cultured for 6 hours. Media were aspirated, and PSCs were washed twice in 50 mL prewarmed PBS and once in 20 mL SF-DMEM. Four mL of SF-DMEM was added and cells were cultured for 6 to 12 hours before CM was harvested and kept on ice. Two mL CM was then added to nonadherent six-well plates, and 5 × 10^5^ neutrophils added to CM and cultured for 6 to 12 hours. Total RNA was extracted using RNeasy Micro Kit (Qiagen, 74004) according to the manufacturer’s instructions.

### Bulk RNA-seq library generation, sequencing, and analysis

For library preparation, cell lysates or RNA were processed according to the previously established SMART-Seq2 protocol (42). Library fragment size distribution and concentration were assessed using Qubit dsDNA HS Assay Kit (Thermo Fisher Scientific, Q32851) and Agilent High Sensitivity DNA Kit (Agilent Technologies, 5067-4626) on a bioanalyzer. Libraries were diluted to 6 nmol/L and pooled, and 20 pmol of pooled libraries were sequenced on Illumina NextSeq 500/550 High Output Kit v2.5 (75 cycles; Illumina, 20024906).

Illumina base call files were converted to FASTQ using the default Illumina bcl2fastq Conversion Software v2.20 pipeline with recommended parameters, and a sample file was constructed with Illumina Experiment Manager. Adapter sequence corresponding to dT30, template switch oligo, as well as Nextera adapter sequence were removed using Cutadapt (3.1, RRID: SCR_011841; ref. 43).

rRNA reads were identified for removal by mapping to 45S (NR_046233.2) and 5S (NR_030686.1) rRNA sequences using bowtie2 (RRID: SCR_016368).

Reads were mapped to the mouse reference genome primary assembly GRCm39 using STAR (35) with default parameters. Duplicated reads were removed in a coverage-dependent manner using a custom script. Briefly, the coverage of exactly identical reads was reduced to that of adjacent sequence using a model based on the Poisson distribution and a cutoff of 0.001 for the probability of the observed coverage.

A custom annotation GTF file was generated from the Ensembl release number 104 annotation by selecting transcripts with protein-coding annotation. The sum of all exons through transcript variants was kept as a single transcript per gene. The read count per exon was derived with HTSeq (RRID: SCR_005514; ref. 44), and the sum of count per gene was calculated as the raw read count. The raw read count was processed for differential gene expression analysis using the DESeq2 package (RRID: SCR_015687; ref. 45).

For analysis of transcription factor activity, aligned BAM files were uploaded for analysis using ISMARA software (46). Sample averaging was performed without batch correction. *Z* values were calculated in RStudio using the averaged delta tables and activity tables derived from ISMARA according to the literature (46). Transcription factors were defined as differentially active in which the *Z*-value is ≥2.

DEGs, identified with DESeq2, were used for GSEA. The R package clusterProfiler (clusterProfiler_4.2.2; ref. 38) was used for GSEA as previously described with the following amendments. Significant DEGs, FC ≥ 1.5 and adjusted *P* value < 0.05, were ranked based on FC and used for the analysis. For Gene Ontology pathway identification, the gseGO function was used from the clusterProfiler R package. Displayed pathways were manually selected from significantly enriched pathways with adjusted *P* value ≤ 0.05.

### Flow cytometry profiling MHCII expression

To assess MHC class II expression in monocultured PSCs, 2.5 × 10^5^ PSCs were seeded in Matrigel domes. Two PSC lines (PSC4 and PSC5) were used in monoculture. Cultures were established in splitting media, mT CM, or splitting media with 100 ρg/mL, 1, 10, or 100 ηg/mL recombinant TGFβ (R&D Systems, 7666-MB-005). For mT CM, mTs were seeded after mechanical fragmentation. mTs were cultured for 2 days in complete murine pancreatic organoid feeding media, and then media were replaced with splitting media and left for 24 hours and CM were collected. After the cultures were established for 24 hours, 100 ρg/mL, 1, 10, or 100 ηg/mL recombinant murine IFNγ was added to either splitting media, CM, or splitting media with equal concentrations of recombinant TGFβ or 100 µmol/L DMXAA. Cultures were dissociated to single cells as described above. For antibody staining, cells were then blocked for nonspecific binding sites for 20 minutes on ice in FB. Antibody staining was conducted using antibodies against MHC class II (Thermo Fisher Scientific) for 30 minutes on ice in SB. Cells were washed in SB and resuspended in LIVE/DEAD fixable Near-IR cell Stain Kit (1:1000 in PBS) for 15 minutes. Cells were profiled on a Bio-Rad ZE5 flow cytometer.

### RT-PCR

2.5 × 10^5^ PSCs were seeded in Matrigel domes in splitting media with 100 µmol/L DMXAA or vehicle (0.5% DMSO) control. Two PSC lines (PSC4 and PSC5) were used in monoculture. After 6 hours, cultures were dissociated as follows: Matrigel domes were gently dislodged with a pipette tip and collected in an excess of splitting media. Cells were spun down and resuspended in 1 mL Cell Recovery Solution (Corning, 354270) on ice for 15 minutes. Cells were spun down, and RNA isolation carried out using RNeasy Mini Kit (Qiagen, 74106) according to the manufacturer’s protocol with on-column DNase treatment. A measure of 500 ng RNA was used for cDNA synthesis using the Thermo Fisher Scientific RevertAid H Minus Reverse Transcriptase (Thermo Fisher Scientific, EP0452) according to the manufacturer’s protocol. 1 µL of cDNA diluted 1:4 was used per qPCR reaction. All primers are listed in Supplementary Table S12. Primers were used at a final concentration of 0.3 µmol/L each. qPCR reactions were performed using PowerUp SYBR Green Master Mix (Thermo Fisher Scientific, A25777) according to the manufacturer’s instructions. The reaction was run in a Quantstudio 6 Flex system. A melting curve was recorded to verify specificity of primers. Gene expression was normalized to *Hprt*. Gene expression was determined with 2^−ΔΔCT^ using QuantStudio Real-Time PCR Software v.1.7.1.

### CXCL10 ELISA on the supernatant

For ELISA, two PSC lines (PSC4 and PSC5) were used in monoculture. A measure of 2.5 × 10^5^ PSCs were seeded in Matrigel domes in splitting media with 100 µmol/L DMXAA or vehicle (0.5% DMSO) control, and the supernatant was collected after 6 hours. CXCL10 concentration in the supernatant was assessed using an ELISA kit (R&D Systems, DY466-05) according to the manufacturer’s protocol. Concentrations were calculated using GraphPad Prism v 8.4.3 with interpolation of a linear standard curve. Spectramax i3x (Molecular Devices) was used for optical density measurements at 450 and 540 nm, in triplicate. Physical imperfections in the plate were corrected by subtracting the optical density at 540 nm from that at 450 nm. Average of triplicate samples was used for quantification.

### Statistical analysis

Statistical analysis and graphical representation were either performed with GraphPad Prism v 8.4.3 (RRID: SCR_002798) or RStudio (RRID: SCR_000432). Statistical tests used are indicated in figure legends.

### Data availability

Publicly available data reanalyzed in this study were obtained from Gene Expression Omnibus GSE197064 and Genome Sequence Archive CRA001160. Mouse sequence data, human sequence data, and code used for analysis are available through EMBL-EBI BioStudies database at https://doi.org/10.6019/S-BSST1896. All other raw data generated in this study are available upon request from the corresponding author.

## Results

### Analysis of mesenchymal subpopulations in PDAC and normal pancreatic stroma defines shared and cancer-specific phenotypes

We utilized scRNA-seq to profile FAP^+^ cell heterogeneity in five primary human PDAC tumors (Supplementary Table S1). We perfpormed FACS to exclude epithelial (EPCAM^+^), immune (CD45^+^), and endothelial (CD31^+^) cells. We positively selected cells expressing FAP, a mesenchymal cell marker (Fig. 1A; ref. 4).

Subsequent quality control steps left 30,786 cells. Clustering at low resolution identified five FAP^+^ populations, each comprised of cells from independent patient samples (Fig. 1B; Supplementary S1A and S1B). Differential expression analysis revealed clusters 0, 1, and 2 had elevated expression of CAF markers (*LUM*, *DCN*, and *COL1A1*; ref. 19). Cluster 3 had elevated expression of perivascular cell markers (*RGS5*, *MCAM*, and *NOTCH3*; ref. 47) and was annotated as cancer-associated perivascular cell (CAP; Fig. 1C; Supplementary Fig. S1C and S1D; Supplementary Table S2). Cluster 4 showed elevated expression of proliferation genes (*TOP2A* and *MKI67*; Fig. 1C).

GSEA revealed fibrotic pathways enriched in CAFs, ECM modulation, and collagen degradation and coagulation, whereas CAPs showed engagement in pathways related to contractility, actin cytoskeletal rearrangements, and Notch signaling (Fig. 1D).

Calculating *Z*-scores for published gene signatures, we observed elevated levels of fibroblast-like CAFs from PDAC (19) and CAFs from breast cancer (47) in our CAF clusters (Fig. 1E and F; Supplementary Table S3). CAPs exhibited elevated signature scores for stellate-like CAFs from PDAC (19) and perivascular-like cells from breast cancer (Fig. 1E and F; ref. 47). Signature scores based on absolute gene expression levels revealed comparable results (Supplementary Fig. S1E and S1F).

We next annotated CAF clusters based on DEGs. Compared with described CAF subtypes, cluster 0 corresponded to ENG^*+*^ CAFs (48), cluster 1 to WT1^*+*^ CAFs (49), and cluster 2 to LRRC15^*+*^ CAFs (Fig. 1G; ref. 18).

We identified tumor regions in tissue sections from the five human PDAC samples profiled by scRNA-seq (Supplementary Fig. S2A). We performed dual stainings with FAP and markers of ENG^+^ (CD105), LRRC15^+^ (COL11A1), and WT1^+^ (CRABP2) CAFs and CAPs (PPP1R14A; Supplementary Table S2). We detected cells that costained for FAP and markers of each subpopulation, confirming their presence in tumor tissue (Fig. 1H; Supplementary Fig. S2B). In a publicly available PDAC scRNA-seq dataset (50), we identified subclusters of mesenchymal cells with elevated *Z*-scores for each CAF subpopulation identified in our data, further validating their presence in PDAC (Supplementary Fig. S2C and S2D).

*ENG* and *WT1* have been described as markers of distinct fibroblast lineages in the healthy pancreas (48, 51). To determine whether FAP^+^ subpopulations identified in our study correspond to mesenchymal subpopulations found in the healthy pancreas, we reanalyzed scRNA-seq data profiling cells from the healthy developing human pancreas (27). First, we extracted mesenchymal cells using author-defined markers to distinguish cell types (Supplementary Table S4). A *de novo* clustering at low resolution identified four mesenchymal cell clusters in the developing pancreas (A, B, C, and D; Fig. 1I; Supplementary Table S4). Cluster A cells expressed pan-CAF markers (*DCN* and *COL1A1*) and heat shock protein family A genes (*HSPA1A* and *HSPA1B*) and showed enrichment in pathways relating to contractility (myogenesis), ECM organization, and collagen deposition (Supplementary Fig. S3A and S3B; Supplementary Table S2). Cluster B was characterized by expression of chemokines (*CXCL1* and *CXCL8*), hyaluronan synthesis genes (*HAS2*), and the stemness-associated gene *CD44* (Supplementary Fig. S3C; Supplementary Table S2) and engagement in IL, TNFα, and Myc signaling pathways (Supplementary Fig. S3B; Supplementary Table S2). Cluster C possessed proliferation associated genes (*TOP2A* and *MKI67*; Supplementary Fig. S3D). Finally, cluster D expressed markers of perivascular cells (*RGS5*, *MCAM*, *ADIRF*, and *CRIP1*) and was enriched for pathways relating to contractility (Supplementary Fig. S3E and S3B; Supplementary Table S2). Interestingly, pathways and genes defining mesenchymal clusters A and B showed features of iCAFs (IL and TNFα signaling) and myCAFs (collagen formation and contractility), respectively, suggesting that mesenchymal cells may exhibit these phenotypes under normal physiologic conditions (Supplementary Fig. S3B). Cluster D shared features with CAPs, suggesting that this population may serve as a cellular reservoir for this subpopulation (Supplementary Fig. S3B).

To determine the similarity of mesenchymal subpopulations in the healthy developing pancreas to those we detect in PDAC, we integrated scRNA-seq data from PDAC and the normal pancreas using label transfer (Fig. 1J) and rPCA (Supplementary Fig. S3F). Both approaches demonstrated that normal mesenchymal subpopulations integrate with specific mesenchymal subpopulations found in PDAC (Fig. 1K; Supplementary S3G–S3J). However, PDAC cluster 2 (LRRC15^+^ CAFs) failed to integrate with cells from the normal pancreas, suggesting that this subpopulation represents a PDAC-specific phenotype (Fig. 1K; Supplementary Fig. S3H). Thus, mesenchymal populations in the healthy pancreas correspond to those observed in PDAC whereas LRRC15^+^ CAFs are only observed in the context of PDAC.

We identified common genes, pathways, and transcription factors between corresponding normal and cancer clusters (Supplementary Fig. S3K–S3M; Supplementary Table S2). Normal A and cancer 0 clusters shared expression of heat shock protein A (*HSPA*) and insulin growth factor–binding protein (*IGFBP*) genes, engagement of the heat shock factor 1 signaling pathway, and activity of SNAI1, RUNX3, and TBX15 transcription factors. Normal B and cancer 1 clusters shared inflammatory markers *CCL2*, *TNFRSF12A*, and *IL1R1*, pathways including TNFα, IL, and Myc signaling, and activity of inflammation, hypoxia, and lipid metabolism–associated transcription factors NFATC2, ARNT, PPARA, and PPARGC1A. Finally, normal D and cancer 3 retained common markers of CAPs, including *RGS5*, *MCAM*, and *CRIP1*, pathways relating to contractility and cytoskeletal rearrangements, and activity of transcription factors associated with muscle contractility, including MYOG.

Together, we define FAP^+^ mesenchymal subpopulations in PDAC and identify their shared and cancer-specific phenotypic characteristics with mesenchymal cell subpopulations of the healthy pancreas (Supplementary Fig. S3N).

### Characterizing FAP^+^ CAF heterogeneity in PDAC identifies iCAF and myCAF subtypes and a novel ifCAF subtype

We next conducted higher-resolution clustering of CAFs (clusters 0, 1, and 2; Fig. 1B), identifying 15 distinct clusters containing cells from independent samples (Fig. 2A; Supplementary Fig. S4A and S4B). We grouped 10 large clusters by CAF subtype markers, *LRRC15* (clusters 0 and 6), *ENG* (clusters 2, 3, 4, 7, and 9), and *WT1* (clusters 1, 5, and 8; Fig. 2B). Cluster 14 also had elevated *WT1* expression. Smaller clusters showed weaker *LRRC15*, *ENG*, or *WT1* expression and were annotated by additional CAF subtype markers, including *PI16* (52) and *VEGFA* (53). Cluster 11 possessed CAP markers, including *MYH11*, and was labeled a CAP-like CAF. A previously undescribed population (cluster 13) expressing interferon-inducible genes including *RSAD2* was identified as ifCAFs.

We defined DEGs within each population (Supplementary Table S5). iCAFs and myCAFs are two well-characterized subtypes in PDAC (4). Four clusters showed elevated expression of myCAF markers, ENG^+^ myCAFs (clusters 4 and 7) and LRRC15^+^ myCAFs (clusters 0 and 6). Similarly, six clusters exhibited elevated expression of iCAF genes, ENG^+^ iCAFs (clusters 2, 3, and 9) and WT1^+^ iCAFs (clusters 1, 5, and 8; Fig. 2C).

We calculated iCAF and myCAF scores in clusters using published gene signatures and pathway gene set signatures (Fig. 2D and E; Supplementary S4C–S4F; Supplementary Table S3; refs. 17, 19). ENG^+^ iCAF clusters 2 and 3 had elevated scores for the Wang and colleagues iCAF signature (19). WT1^+^ iCAF clusters 1 and 5 showed high iCAF scores for the Elyada and colleagues iCAF signature (17). myCAF clusters demonstrated low iCAF scores, except cluster 7, which was modestly enriched for one signature (Fig. 2D). LRRC15^+^ myCAF clusters and ENG^+^ myCAF cluster 7 had high myCAF scores for both myCAF signatures, whereas cluster 4 showed an increased score for the Elyada and colleagues myCAF signature (Fig. 2E). Thus, myCAF subtypes in our data match those in previous studies, whereas iCAFs defined by Wang and colleagues and Elyada and colleagues correspond to distinct ENG^+^ and WT1^+^ iCAF subtypes, respectively. In addition, metabolically active CAFs identified by Wang and colleagues align with iCAFs described by Elyada and colleagues and our WT1^+^ iCAFs (Supplementary Fig. S4D). Amongst the smaller clusters, only ifCAFs, with low iCAF and high myCAF scores, demonstrated a clear iCAF or myCAF phenotype (Supplementary Fig. S4E and S4F).

iCAF and myCAF subtypes were present in all samples, but their proportion varied significantly (Supplementary Fig. S4G). Samples 1, 2, 3, and 6 had a majority ENG^+^ iCAFs and myCAFs, sample 4 a majority of LRRC15^+^ myCAFs, and sample 5 had mainly WT1^+^ iCAFs. CAF subtype prevalence has been reported to vary in tumors with dense and loose desmoplasia (19). We found no striking differences between tumors with dense and moderate/loose desmoplasia (Supplementary Fig. S4H).

We assessed the activity of transcription factors associated with iCAFs and myCAFs (Fig. 2F) and identified differentially active transcription factors between iCAF and myCAF subtypes (Supplementary Fig. S4I; Supplementary Table S5). TGFβ signaling transducers SMAD3 and SMAD4, key drivers of myCAF formation (7), exhibited high activity in LRRC15^+^ myCAFs, whereas ENG^+^ myCAFs displayed lower levels. SMAD3 and SMAD4 activity was repressed in ENG^+^ iCAFs but enriched in WT1^+^ iCAFs. Additional profibrotic transcription factors RUNX2, TWIST1, and LEF1 were active in LRRC15^+^ myCAFs. ENG^+^ myCAFs showed elevated histone deacetylase 3 activity, linked to promoting fibrosis (54). Myogenic factor 5 was active in both myCAF subtypes. Hedgehog signaling transducers GLI1 and GLI2, implicated in myCAF differentiation (55), were not strongly enriched in iCAF or myCAF subtypes. Inflammatory transcription factors NFKB1 and STAT3 had higher activities in WT1^+^ iCAFs than ENG^+^ iCAFs, although both iCAF subtypes had higher activities than myCAFs. Hypoxia is also associated with an iCAF phenotype (56), and hypoxia response transcription factors HIF1A and ARNT were differentially active in WT1^+^ iCAFs, suggesting that WT1^+^ iCAFs are hypoxic. SMAD6 and SMAD7 were more active in WT1^+^ iCAFs and ENG^+^ iCAFs, respectively. SMAD7 potently inhibits TGFβ signaling, whereas SMAD6 has been shown to be a weaker repressor (57); this may account for higher levels of TGFβ signaling in WT1^+^ iCAFs and stronger repression in ENG^+^ iCAFs.

GSEA demonstrated both myCAF subtypes had engagement of canonical myCAF-associated pathways, including collagen formation, ECM organization, and myogenesis (Supplementary Fig. S4J and S4K; Supplementary Table S5). LRRC15^+^ myCAFs were enriched in activin signaling, striated muscle contraction, and IL signaling, whereas ENG^+^ myCAFs exhibited laminin interactions and Notch3 and hedgehog signaling. Both iCAF subtypes shared JAK/STAT signaling and complement cascades pathways. WT1^+^ iCAFs possessed TNFα and Myc signaling, the inflammatory response, and hypoxia, alongside canonical myCAF-associated pathways. ENG^+^ iCAFs were defined by IL7 signaling, IFNα response, inflammasomes, and senescence-associated secretory phenotype pathways.

Among the smaller clusters, VEGFA^+^ CAFs possessed proangiogenic markers, including *VEGFA* and *LOX* (Supplementary Fig. S5A). WT1^+^ CAFs were distinguished from WT1^+^ iCAFs through reduced expression of iCAF markers and *EPYC* expression (Supplementary Fig. S5B). PI16^+^ CAFs expressed ECM components, including *SBSPON* and *PRG4*, whereas CAP-like CAFs expressed CAP markers, in addition to CAF markers, including *RGS5* and *MCAM* (Supplementary Fig. S5C and S5D).

ifCAFs were characterized by genes (*RSAD2*, *IFIT3*, *CXCL10*, and *DDX58*), pathways (interferon signaling), and transcription factors (IRF1/2/3/9) involved in the interferon response (Fig. 2G–I). To validate ifCAFs’ presence in tissue, we performed multiplex *in* situ hybridization. CAFs and tumor cells were identified using *DCN* and *KRT19*, respectively. A subset of CAFs were shown to be positive for the interferon-response genes *RSAD2* and *IFIT3* (Fig. 2J).

In a publicly available PDAC scRNA-seq dataset (50), subclustering CAFs at a high resolution showed that all CAF clusters expressed *FAP* (Supplementary Fig. S5E and S5F). All subtypes present in our data could be identified in the Peng and colleagues dataset through signature *Z*-scores, except for two rare populations, WT1^+^ CAFs and CAP-like CAFs (Supplementary Fig. S5G).

Taken together, we characterize iCAF and myCAF subtypes and show ifCAFs represent a novel CAF subtype in PDAC defined by an interferon-response phenotype.

### Tumor cell–derived signals induce iCAF, myCAF, and ifCAF phenotypes in a three-dimensional *in vitro* coculture model of PDAC

**Figure 3.**
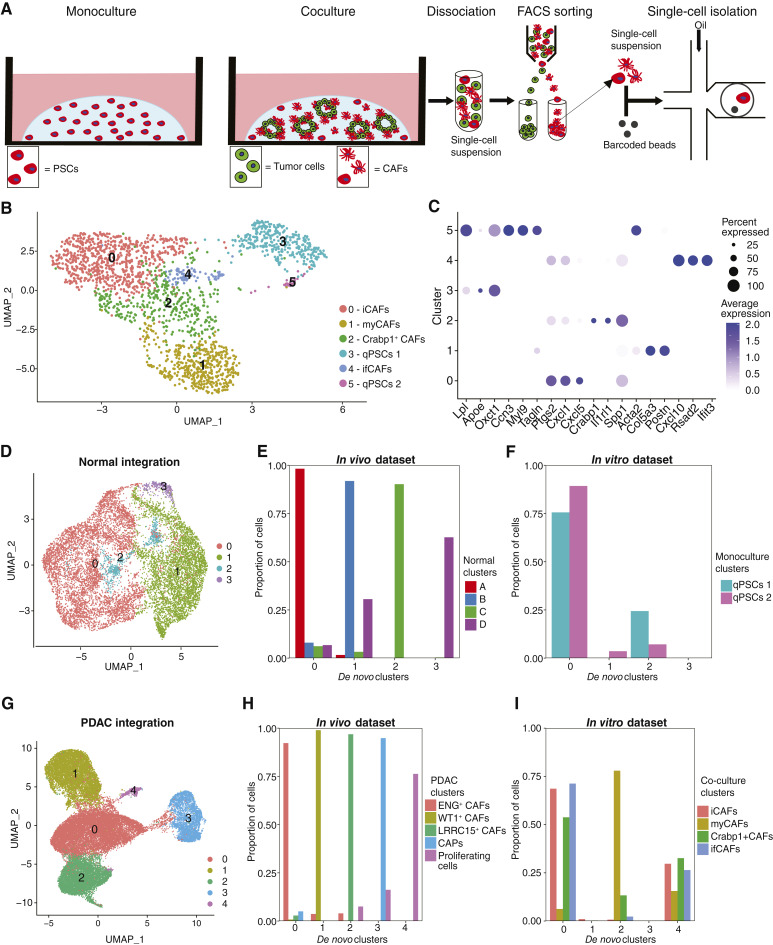
An *in vitro* murine coculture model system can recapitulate CAF heterogeneity observed *in vivo*. **A,** Workflow illustration for profiling PSCs in monoculture or coculture with tumor organoids. PSCs were isolated from monoculture or coculture using FACS before droplet-based scRNA-seq. **B,** Unsupervised clustering of FACS-sorted single cells for 11 cDNA libraries (two monoculture and nine coculture) derived from two PSC cell lines (PSC4 and PSC5) visualized through UMAP. **C,** Dot plot displaying DEG markers of PSC and CAF subtypes. The size of dots represents the percentage of cells in the cluster in which gene expression is detected. Color denotes scaled expression values. **D,** Unsupervised clustering of integrated scRNA-seq data from mesenchymal cells (normal pancreas) *in vivo* from Fig. 1H and qPSC clusters *in vitro* from **B** (clusters 3 and 5) visualized through UMAP. **E** and **F,** Bar plots quantifying proportion of cells from mesenchymal subpopulations (normal pancreas) *in vivo* (**E**) and qPSC subtypes from *in vitro* (**F**) in *de novo* clusters. **G,** Unsupervised clustering of integrated scRNA-seq data from FAP^+^ mesenchymal cells (PDAC) *in vivo* from Fig. 1B and cocultured CAF clusters *in vitro* from **B** (clusters 0, 1, 2, and 4) visualized through UMAP. **H** and **I,** Bar plots quantifying proportion of cells from mesenchymal subpopulations (PDAC) *in vivo* (**H**) and PSC-derived CAF subtypes from *in vitro* (**I**) in *de novo* clusters.

To test whether tumor cell–derived signals are sufficient to induce differentiation of CAF subtypes, we used a three-dimensional coculture model involving fluorescently labeled PSCs, a defined cellular reservoir of CAFs (4), and pancreatic tumor organoids, obtained from the KPC mouse model (20). We isolated PSCs by FACS for scRNA-seq profiling, which yielded 2,008 single cells after quality control (Fig. 3A; Supplementary S6A). We identified six clusters, comprising cells from both biological replicates (Fig. 3B; Supplementary Fig. S6B and S6C). Quiescent PSCs (qPSC) in monoculture differed from those activated to become CAFs in coculture (Supplementary Fig. S6D).

We identified cluster 0 as iCAFs based on expression of inflammatory genes (*Ptgs2*, *Cxcl2*, and *Cxcl5*) and engagement of inflammatory pathways and transcription factors, including STAT3 and NFκB1 (Fig. 3C; Supplementary S6E and S6F; Supplementary Table S6). Cluster 4 shared these inflammatory features but was identified as ifCAFs through detection of genes (*Cxcl10*, *Rsad2*, and *Ifit3*), pathways (IFN signaling), and transcription factors (Irf1/2/3/7/9) relating to the IFN response. Cluster 1 exhibited a myofibroblastic phenotype possessing markers (*Acta2*, *Col5a3*, and *Postn*) and pathways relating to contractility and actin cytoskeletal rearrangements and was designated a myCAF. myCAFs also exhibited higher activity of the TGFβ signal transducer SMAD3. qPSC clusters showed elevated expression of genes (*Lpl*, *Apoe*, and *Oxct1*), activity of transcription factors (EBF1 and SOX9), and pathway enrichments (cluster 3 only) related to lipid metabolism and storage (58, 59). Cluster 2 exhibited fewer DEGs and was labeled a Crabp1^+^ CAF based on its top marker. Crabp1^+^ CAFs were enriched in translation-associated pathways and possessed high MYC activity.

iCAF, ifCAF, and myCAF signatures from *in vitro* cocultures showed elevated scores in corresponding CAF subtypes *in vivo* (Supplementary Fig. S6G–S6I). The *in vitro* myCAF score was also high in WT1^+^ iCAF clusters 1 and 5, which could be a consequence of the elevated TGFβ signaling activity in WT1^+^ iCAFs (Supplementary Fig. S6I). The Crabp1^+^ CAF coculture signature was higher in WT1^+^ iCAFs (Supplementary Fig. S6J). Thus, *in vitro*–generated CAF subtypes demonstrated iCAF, ifCAF, and myCAF phenotypes consistent with those observed *in vivo.*

To examine similarities between qPSCs in our *in vitro* model and mesenchymal subpopulations in the healthy pancreas, we integrated the qPSC clusters (3 and 5; Fig. 3B) with mesenchymal cells from the normal developing pancreas (Fig. 1H; ref. 27), yielding four clusters (Fig. 3D). We quantified the proportion of cells within *de novo* clusters following integration based on cell annotation in the separate datasets. The majority of *in vitro* qPSCs integrated with cells from *in vivo* mesenchymal cluster A (myofibroblastic phenotype; Fig. 3E and F). In contrast, a tiny fraction of *in vitro* qPSCs integrated with mesenchymal cluster B (inflammatory phenotype) and none integrated with mesenchymal cluster D (perivascular cells). This demonstrates that PSCs represent a specific subpopulation of mesenchymal cells in the healthy pancreas with a myofibroblastic phenotype.

To determine whether PSC-derived CAFs formed *in vitro* related to CAF subtypes defined *in vivo,* we integrated *in vivo* iCAF, myCAF, ifCAF, and Crabp1^+^ CAF clusters (0, 1, 2, and 4; Fig. 3B) with FAP^+^ mesenchymal cells in PDAC (Fig. 1B), identifying five clusters (Fig. 3G). Cluster 0 contained ENG^+^ CAFs from *in vivo* (Fig. 3H) and the majority of iCAFs, ifCAFs, and Crabp1+ CAFs from *in vitro* (Fig. 3I). The majority of *in vitro* myCAFs were present in cluster 2, which contained the cancer-specific LRRC15^+^ CAFs from *in vivo* (Fig. 3H and I). A significant proportion of all *in vitro* CAF subtypes were present in cluster 4, which contained proliferating cells from *in vivo* (Fig. 3H and I). In contrast, only a tiny fraction of iCAFs were present in cluster 1, which possessed WT1^+^ CAFs from *in vivo*. In addition, no CAFs from *in vitro* were present in cluster 3, which was composed of CAPs from *in vivo* (Fig. 3H and I).

In summary, we demonstrate that the *in vitro* coculture model can generate iCAFs, myCAFs, and ifCAFs from qPSCs in response to tumor-derived signals. Furthermore, we show that our *in vitro*–cultured PSCs correspond to a specific subpopulation of mesenchymal cells in the healthy pancreas with a myofibroblastic phenotype. Amongst CAFs derived from PSCs *in vitro*, iCAFs and ifCAFs align with *in vivo* ENG^+^ CAFs and myCAFs align with the LRRC15^+^ CAFs subpopulation. No *in vitro* CAFs corresponded to CAPs or WT1^+^ CAFs observed in PDAC.

### A time-course profiling CAF differentiation *in vitro* through scRNA-seq elucidates the dynamics of CAF subtype formation

We used scRNA-seq to characterize the differentiation of PSCs during coculture with tumor cells at a series of time points. After quality control, we retained 3,458 cells.

Initial *de novo* clustering trajectory analysis, using Slingshot, revealed seven clusters (Supplementary Fig. S7A–S7F; ref. 37). However, pseudotime did not align with chronologic time, likely due to the significant transcriptional difference between day 0 and day 1 cells, which was largely driven by engagement of pathways relating to translation (Supplementary Fig. S7G). This prevented analysis of the full differentiation trajectory from day 0 to day 6.

**Figure 4.**
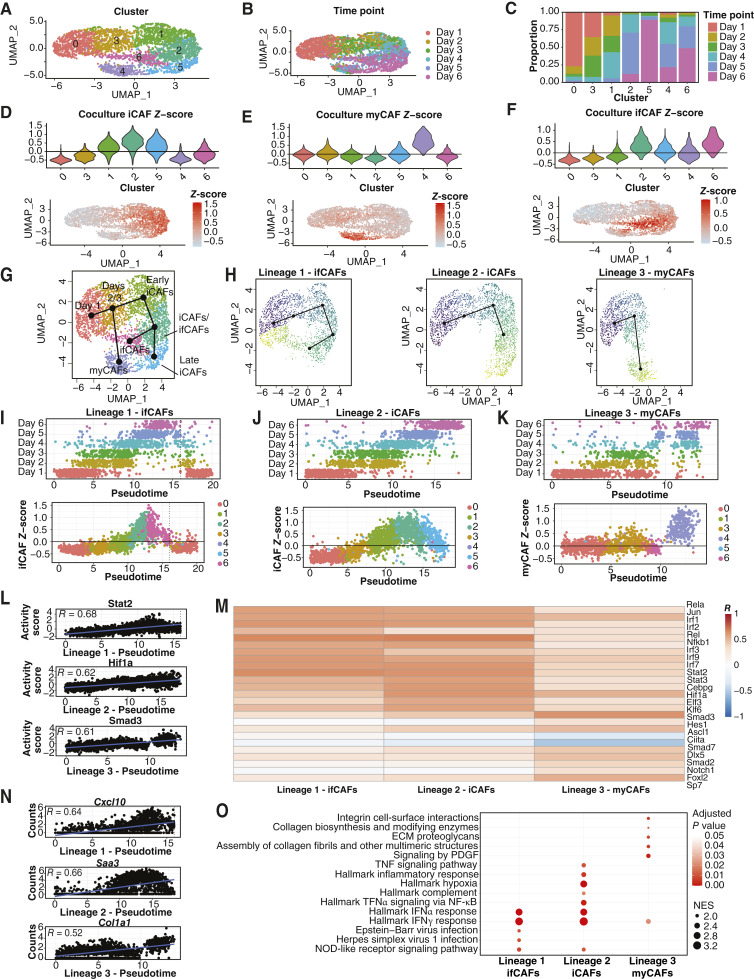
A time-course profiling CAF differentiation *in vitro* through scRNA-seq elucidates the dynamics of CAF subtype formation. **A** and **B,** Unsupervised clustering of FACS-sorted single cells for 13 cDNA libraries from 6 time points (PSC5 + mT3) visualized through UMAP. Cells colored by cluster (**A**) or time point (**B**). **C,** Bar plots displaying proportion of cells within clusters from time points. Colored by time point. **D–F,** Violin (top) and UMAP (bottom) plots depicting *Z*-scores of gene signatures of myCAFs (**D**), iCAFs (**E**), and ifCAFs (**F**) as defined in Fig. 3B. **G,** UMAP plot (**A**) showing trajectory of CAF differentiation in coculture inferred by Slingshot. **H,** UMAP plot (**A**) split by lineage and colored by pseudotime inferred by Slingshot. **I–K,** Top, scatter plots showing cell pseudotime values across time points for each lineage. Bottom, scatter plots showing cell pseudotime values against CAF subtype signature scores for each lineage. Dotted line in **I** indicates pseudotime cutoff for inclusion, see Materials and Methods. **L,** Scatter plots showing the top correlated transcription factor activities against pseudotime values for each lineage. **M,** Heatmap of the correlation between transcription factor activity and pseudotime values for the top 10 differentially correlated transcription factors in each lineage. Color scale indicates the Pearson product moment correlation coefficient. **N,** Scatter plots showing the top correlated gene normalized counts against pseudotime values for each lineage. **O,** GSEA showing enriched Hallmark, KEGG, and Reactome pathways in lineages. Selected pathways visualized in a dot plot, in which the size of dots represents the FC in pathway enrichment and the color displays the *q* values of the pathways. NES, normalized enrichment score.

To address this, we repeated the analysis, omitting day 0 and instead rooting the trajectory in the day 1 cluster. We identified seven clusters, finding clustering was only partially driven by time-point (Fig. 4A–C) and not by replicates (Supplementary Fig. S7H and S7I). Based on signature *Z*-scores from the coculture model described above, clusters 1, 2, and 5 exhibited iCAF characteristics, indicating early iCAF formation (cluster 1) and later maturation of iCAFs (clusters 2 and 5; Fig. 4D). Cluster 4 had a pronounced myCAF score and contained mostly cells from days 4, 5, and 6, indicating myCAF formation occurs later in coculture (Fig. 4E). Cluster 6 had an elevated ifCAF signature score along with cluster 2, which also had a high iCAF score, suggesting an intermediate phenotype. The ifCAF cluster 6 was formed of cells mainly from days 5 and 6, signifying ifCAFs also form later in coculture (Fig. 4F). The Crabp1^+^ CAF signature was enriched in cluster 0, containing mainly cells from day 1 of coculture, reaffirming the increased translational activity of this cluster (Supplementary Fig. S7J).

We generated trajectories with Slingshot, segmenting differentiation into three lineages; ifCAFs (lineage 1), iCAFs (lineage 2), and myCAFs (lineage 3; Fig. 4G and H). Pseudotime ordering aligned with chronologic time, and mature subtype signature scores increased with pseudotime (Fig. 4I–K).

We correlated inferred transcription factor activities with pseudotime for each lineage and identified the top correlated transcription factors and effector genes (Fig. 4L–N; Supplementary Table S8). Transcription factor activities for the ifCAF lineage and iCAF lineage were similarly correlated with pseudotime likely because both lineages pass through the early iCAF cluster 1 and the iCAF/ifCAF intermediate cluster 2. Transcription factors, which regulate the IFN response, including IRF1/2/3/7/9 and STAT2, were positively correlated in both lineages. Similarly, inflammatory transcription factors JUN, REL, RELA, NFκB1, and STAT3 activity increased across pseudotime in both lineages although STAT3 was more associated with the iCAF lineage. HIF1A was also more strongly correlated with iCAF differentiation, suggesting that this lineage becomes more hypoxic over time. The TGFβ signaling transducer SMAD3 was strongly correlated with pseudotime in the myCAF lineage, and the TGFβ signaling inhibitor SMAD7 was inversely correlated, again highlighting the role of TGFβ in myCAF differentiation. An unknown juxtacrine signaling component has also been associated with myCAF differentiation (4). Interestingly, we find the NOTCH1 juxtacrine signaling pathway and downstream effector HES1 to be correlated with myCAF differentiation, providing evidence that this is the undefined juxtacrine component driving myCAF differentiation. GSEA identified pathways engaged during subtype differentiation (see Materials and Methods; Supplementary Table S8). Corroborating the identity of the ifCAF lineage, we identified pathways involved in IFN response in lineage 1 (Fig. 4O). interferon-response pathways were shared in the iCAF lineage 2, but additional pathways, including TNFα signaling, complement, inflammatory response, and hypoxia, were associated with this lineage specifically. Finally, myCAF differentiation was associated with activation of pathways relating to collagen formation and PDGF signaling.

In summary, iCAFs form earliest in response to tumor-derived signals whereas ifCAFs and myCAFs form after a longer period of coculture. Inflammatory and interferon-response pathways and transcription factors were present in iCAF and ifCAF differentiation, but iCAFs demonstrated engagement of additional canonical inflammatory pathways. We reaffirm the role of TGFβ in driving myCAF differentiation and uncover a potential role for Notch1 signaling as a juxtacrine signaling component associated with myCAF differentiation.

### Type I and type II IFN pathways govern ifCAF and apCAF formation, respectively

To characterize the induction of IFN response in ifCAFs, we examined the ability of type I or type II IFN signaling agonists to induce ifCAF and apCAF phenotypes. PSCs treated with the stimulator of IFN gene (STING) agonist DMXAA, which promotes type I IFN signaling, exhibited strong upregulation of the IFN response and inflammatory genes and elevated protein secretion of the ifCAF marker CXCL10 (Supplementary Fig. S8A and S8B). Transcriptional profiling after DMXAA treatment for 6 hours in monoculture and coculture showed a significant increase in ifCAF scores but no other CAF subtype (Supplementary Fig. S8C–S8H).

To test the ability of type II IFN signaling to induce apCAFs, defined by MHCII expression, in PSCs, we cultured PSCs in monoculture or coculture for 4 days before IFNγ addition for a further 2 days and assessed MHCII expression by flow cytometry (Supplementary Fig. S8I). IFNγ significantly increased the proportion of MHCII^+^ PSCs in both monoculture and coculture, although MHCII induction was significantly higher in monoculture (56.2%) than coculture (6.78%). Addition of IFNγ to coculture from day 0 induced MHCII expression in a proportion of PSCs similar to monoculture, suggesting prior exposure to tumor-derived signals suppresses IFNγ-mediated MHCII expression (Supplementary Fig. S8J).

Activated T cells from C57BL/6J mice spleens added to the media surrounding PSCs, increased MHCII^+^ PSCs in monoculture but not coculture (Supplementary Fig. S8K). PSCs in monoculture treated with tumor cell CM or recombinant TGFβ were both able to significantly suppress MHCII induction by IFNγ, supporting previous findings (Supplementary Fig. S8L; ref. 48). Thus, apCAFs can be formed from PSCs through treatment with IFNγ or exposure to activated T-cells but diffusible factors from tumor cells suppress apCAF formation.

We profiled PSCs treated with DMXAA or IFNγ-induced apCAFs by flow cytometry and bulk RNA-seq. We found that only apCAFs expressed MHCII and were specifically enriched for pathways and transcription factor activity relating to MHCII antigen presentation, though both conditions shared an interferon-response phenotype (Supplementary Fig. S8M–S8O; Supplementary Table S9). Thus, STING agonists induce ifCAFs, through type I IFN, whereas IFNγ promotes apCAFs via type II IFN signaling.

### STING agonism reprograms the TME and suppresses invasive properties in a mouse model of PDAC

**Figure 5.**
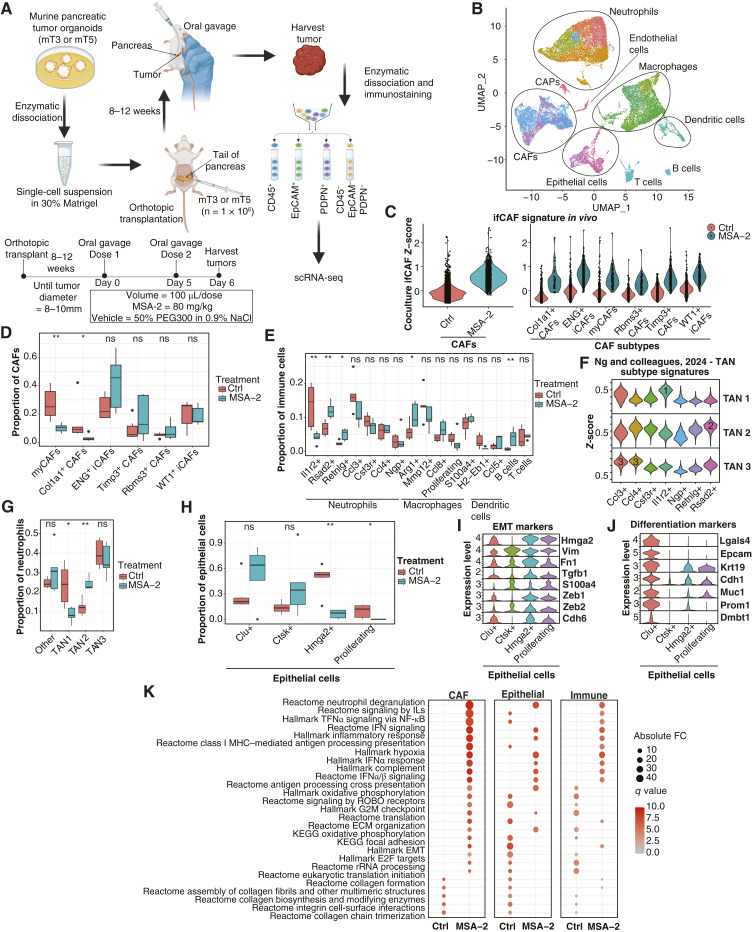
STING agonism reprograms the TME and suppresses invasive properties in a mouse model of PDAC. **A,** Schematic illustration of MSA-2 treatment in orthotopically transplanted tumors. Tumor organoid lines (mT3 or mT5) were injected into the tail of the pancreas in mice. Once the tumor had reached 8–10 mm (8–12 weeks), MSA-2 (80 mg/kg) or vehicle control was administered by oral gavage; two doses were given 5 days apart, and the tumor was harvested 6 days after the initial dose. Cell populations were isolated by FACS, pooled equally, and profiled with scRNA-seq. **B,** Unsupervised clustering of single cells for 11 cDNA libraries derived from 3 MSA-2–treated and 3 control PDAC specimens visualized through UMAP. **C,** Violin plots depicting *Z*-scores of gene signatures of ifCAFs as defined in Fig. 3B. **D** and **E,** Bar plots of CAF subtype (**D**) and immune subtype (**E**) proportions in control and MSA-2–treated tumors. **F,** Violin plots of TAN subtype signature *Z*-scores from Ng and colleagues (62). **G,** Bar plots showing TAN subtype proportions in control and MSA-2–treated tumors. **H,** Bar plots of epithelial subtype proportions in control and MSA-2–treated tumors. **I–J,** Stacked violin plots showing normalized gene expression levels of EMT-associated (**I**) and differentiation-associated (**J**) genes in epithelial clusters. **K,** GSEA showing enriched Hallmark, KEGG, and Reactome pathways in immune, CAF, and epithelial compartments between control and MSA-2–treated tumors. Selected pathways visualized in a dot plot, in which the size of dots represents the FC in pathway enrichment and the color displays *q* values of the pathways. Conditions are compared through the Wilcoxon rank-sum test (**D, E, G,** and **H**). ns, not significant; *, *P* < 0.05; **, *P* < 0.01. **A,** Created in BioRender. Dongre, M. (2025) https://BioRender.com/0ix9jah.

STING agonists have demonstrated therapeutic benefits in murine models of PDAC through enhancing antitumor immunity (60, 61). However, their influence on CAFs has not been well defined. To address this, we orthotopically transplanted PDAC organoids into mice and treated with an orally available STING agonist (MSA-2) before profiling tumors with scRNA-seq (Fig. 5A).

We identified 27 clusters that were classified as cell types based on canonical markers (Supplementary Fig. S9A). Where multiple clusters were present for a single cell type, clusters were labeled based on the top DEG (Fig. 5B; Supplementary Table S10). CAF subtypes were classified based on gene signature scores from our *in vivo* and *in vitro* datasets (Supplementary Fig. S9B and S9C). We identified a myCAF cluster, ENG^+,^ and WT1^+^ iCAFs, in addition to three clusters that were not strongly enriched for any signature and labeled as Timp3^+^, Col1a1^+^, and Rbms3^+^ CAFs. Clustering was not driven by the cells’ origin from control versus MSA-2–treated tumors (Supplementary Fig. S9D). Whereas we did not identify an ifCAF cluster at this resolution, cells with high ifCAF signature scores were present in control tumors and ifCAF signature scores were significantly elevated across all CAF clusters following MSA-2 treatment (Fig. 5C). We found that treating with MSA-2 led to a significant reduction in the proportion of myCAFs alongside Col1a1^+^ CAFs (Fig. 5D).

Amongst immune cells, MSA-2 treatment significantly increased the proportion of Arg1^+^ macrophages and B cells (Fig. 5E). Notably, we found that MSA-2 had a pronounced effect on neutrophil populations with a significant reduction in Ilr2^+^ neutrophils and a significant increase in Retnlg^+^ and Rsad2^+^ neutrophils. Signatures scores of the previously described neutrophil subtypes from Ng and colleagues (62) revealed some of the clusters in our dataset correspond to TAN subtypes, TAN1, TAN2, and TAN3 (Fig. 5F). MSA-2 significantly reduced the proportion of the TAN1 subtype while significantly increasing the proportion of the TAN2 subtype (Fig. 5G). Type I IFN signaling, associated with an antitumor phenotype in TANs (63, 64), was globally increased in TANs following MSA-2 treatment and highest in the TAN2 subtype, which was more prevalent following MSA-2 treatment (Supplementary Fig. S9E). STING agonists have been reported to enhance antitumor immunity through enhancing T-cell activity (60). Whereas there was no significant increase in T-cell proportion, CD8 T-cell activation and cytolytic signature scores were significantly elevated following STING agonism (Supplementary Fig. S9F).

MSA-2 strongly affected epithelial cells with a significant decrease in the proportion of Hmga2^+^ and proliferating epithelial cells (Fig. 5H). A nonsignificant but pronounced increase was observed in Clu^+^ epithelial cells, the dominant cluster in MSA-2–treated tumors. The Hmg2a^+^ cluster exhibited elevated levels of epithelial-to-mesenchymal transition (EMT) markers compared with the Clu^+^ epithelial cluster (Fig. 5I). In contrast, the Clu^+^ cluster had elevated expression of markers for differentiated epithelial cells (Fig. 5J).

GSEA revealed a depletion of myCAF associated pathways relating to collagen deposition and ECM regulation upon MSA-2 treatment (Fig. 5K), in agreement with the reduced number of myCAFs. MSA-2 suppressed EMT and proliferative pathways in epithelial cells, whereas in immune cells, MSA-2 suppressed pathways relating to translation. CAFs, epithelial cells, and immune cells were all enriched for interferon-response and proinflammatory pathways in MSA-2–treated tumors. Taken together, we found that treating tumor-bearing mice with a STING agonist induced CAFs with a more pronounced ifCAF phenotype at the expense of the myCAF population, gave rise to a polarization of TANs toward an antitumor phenotype, and led to the suppression of EMT in tumors.

### STING agonism in CAFs promotes type I IFN signaling and polarization of TANs toward an antitumor phenotype

**Figure 6.**
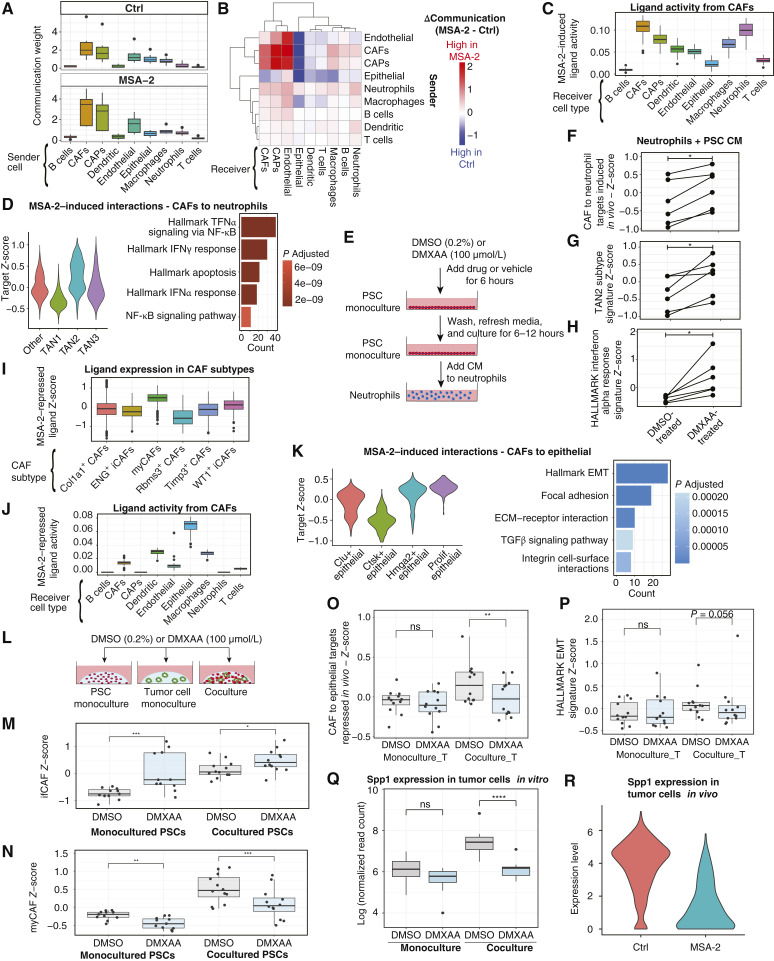
STING agonism in CAFs promotes TAN polarization and suppresses their prometastatic influence on tumor cells. **A,** Box plots showing communication weights, inferred using CellChat, of cell types in control (top) and MSA-2 (bottom)–treated tumors. **B,** Heatmap showing differences in communication weights between sender and receiver cell types in control and MSA-2–treated tumors (Ctrl – MSA-2). **C,** Activity scores, inferred using NicheNet, for MSA-2–induced ligands in CAFs and receiver cell types. **D,** MSA-2–induced CAF ligand targets in TANs, identified using NicheNet. Left, *Z*-score of target gene expression in TAN subtypes. Right, overrepresentation analysis of target genes. **E,** Schematic illustration of workflow. PSCs treated with DMSO (0.2%) or DMXAA (100 μmol/L) for 6 hours, thoroughly washed to remove drug, cultured in fresh media for 6 to 12 hours, CM harvested and added to bone marrow–derived primary neutrophils for 6 to 18 hours. RNA was extracted from neutrophils before RNA-seq. **F–H,***Z*-Scores for gene signature expression in bone marrow–derived primary neutrophils exposed to CM from PSCs treated with DMSO or DMXAA (100 μmol/L). STING agonist induced CAF ligand targets in neutrophils defined *in vivo* (**F**), TAN two subtype signature (**G**), Hallmark IFN response signature (**H**). Points represent paired replicates, CM derived from PSC4 (*n* = 3) and PSC5 (*n* = 3). **I,** Box plots showing *Z*-scores of MSA-2–repressed ligand expression in CAFs between CAF subtypes. **J,** Activity scores, inferred using NicheNet, for MSA-2 repressed ligands in CAFs and receiver cell types. **K,** MSA-2–repressed CAF ligand targets in epithelial cells identified using NicheNet. Left, *Z*-score of target gene expression in epithelial clusters. Right, overrepresentation analysis of target genes. **L,** Schematic of experimental conditions. DMSO (0.2%) and DMXAA (100 µmol/L) added to PSC and tumor cell monocultures and cocultures before FACS isolation of cell types and bulk RNA-seq. **M–P,***Z*-scores for gene signature expression in PSCs and tumor cells in monoculture and coculture treated with DMSO and DMXAA (100 µmol/L). **M** and **N,** ifCAF (**M**) and myCAF (**N**) signatures from Fig. 3. **O,** STING agonist repressed CAF ligand targets in epithelial cell defined *in vivo* (**K**). **P,** Hallmark EMT signature. Points represent paired replicates, monocultures PSC4 (*n* = 5), PSC5 (*n* = 6), mT3 (*n* = 6), mT4 (*n* = 6), and cocultures PSC4 + mT4 (*n* = 6) and PSC5 + mT3 (*n* = 6). **Q,** Box plots showing normalized expression of *Spp1* in monocultured and cocultured tumor cells. **R,** Violin plots showing *Spp1* expression in epithelial cells in orthotopically transplanted tumors *in vivo*. Samples are compared through paired Wilcoxon rank-sum tests (**F–H** and **M–P**) and the Wald test from the DESeq2 package (**Q**). ns, not significant; *, *P* < 0.05; **, *P* < 0.01; ***, *P* < 0.001; ****, *P* < 0.0001.

We used CellChat (32) to predict signaling interactions between cell types in control and MSA-2–treated tumors. In both conditions, CAFs were predicted to be the strongest interactors (Fig. 6A). Communication between nonimmune stromal cell types (CAFs, CAPs, and endothelial cells) was enhanced by MSA-2 (Fig. 6B). CAF interactions with macrophages, B cells, and neutrophils were also elevated in response to drug treatment. Interactions between epithelial cells and other cell types were generally repressed by MSA-2, including communication from CAFs to epithelial cells.

Given that CAFs were the most interactive cell type and that MSA-2 modified their communication with cell populations, which were strongly affected by MSA-2 treatment (tumor cells, B cells, macrophages, and neutrophils; Fig. 5), we utilized NicheNet (33) to identify potential ligand–target interactions between CAFs and other cell populations. We defined ligands in CAFs and their targets in cell types that were induced or repressed by MSA-2 (Supplementary Table S10). Ligands induced by MSA-2 in CAFs were most highly expressed in iCAF subtypes (Supplementary Fig. S10A) and demonstrated the highest activity on neutrophils (Fig. 6C). Interestingly, targets of CAF ligands that were induced in neutrophils in MSA-2–treated tumors were highly expressed in the TAN2 subtype (Fig. 6D, left). We found that inflammatory, apoptotic, and interferon-response pathways were overrepresented in these targets, suggesting that CAFs may augment the direct effects of the drug (Fig. 6D, right).

To interrogate the direct influence of ifCAFs on TANs, we treated PSCs *in vitro* with the STING agonist DMXAA, which induces an ifCAF phenotype, or DMSO control (Supplementary Fig. S6). Neutrophils, FACS-purified from bone marrow (Supplementary Fig. S10B), were then exposed to drug-free CM derived from DMSO- or DMXAA-treated PSCs before profiling with bulk RNA-seq (Fig. 6E). Neutrophils exposed to CM from DMXAA-treated PSCs showed induction of type I interferon-response genes (Supplementary Fig. S10C). Furthermore, we found that targets of CAF ligands that were induced in neutrophils by STING agonists *in vivo* were significantly elevated in the neutrophils exposed to ifCAF CM, reaffirming the ligand–target interactions defined *in vivo* (Fig. 6F). Neutrophils exposed to ifCAF CM also showed significantly higher signature scores for type I IFN signaling and the TAN2 subtype but no significant differences in other TAN subtypes (Fig. 6G and H; Supplementary Fig. S10D). Thus, ifCAFs can directly promote TAN polarization toward a TAN2 phenotype and stimulate type I IFN signaling in TANs, which is associated with an antitumor phenotype.

### STING agonism in CAFs suppresses the prometastatic influence of CAFs on tumor cells

Our data above suggest a model whereby STING agonism suppresses tumor cell proliferation and EMT characteristics. We next investigated whether the impaired signaling between CAFs and epithelial cells following MSA-2 treatment (Fig. 6B) could account for this. Ligands repressed by MSA-2 in CAFs were highly expressed in myCAFs (Fig. 6I) and had the highest activity in CAF to epithelial communication (Fig. 6J). Targets of CAF ligands that were repressed in epithelial cells by MSA-2 were highly expressed by Hmga2^+^ and proliferating epithelial cells (Fig. 6K, left). Also, overrepresentation analysis of CAF ligand targets, which were suppressed in epithelial cells by MSA-2, identified pathways associated with EMT (Fig. 6K, right). Thus, our data suggest that STING agonism represses the production of CAF-derived signals that promote EMT in tumor cells.

Because ifCAF induction supports a more antitumoral microenvironment, we next sought to examine whether STING agonists could synergize with chemotherapeutic agents. We treated tumor cells in monoculture or coculture with PSCs over a time course with gemcitabine, DMXAA, or a combination treatment of the two. We observed a synergistic effect of DMXAA and gemcitabine on tumor cell growth, though this effect was CAF independent, as demonstrated by the reduction in tumor cell covered area in both monoculture and coculture (Supplementary Fig. S11A).

To further examine the direct effect of ifCAFs on tumor cells, we treated PSCs and tumor cells in monoculture and coculture with the STING agonist DMXAA (Fig. 6L), now for 24 hours, before isolating PSCs and tumor cells using FACS for profiling with bulk RNA-seq. DMXAA treatment of PSCs in monoculture and coculture resulted in a significant increase in ifCAF signature scores and a significant decrease in myCAF signature scores, recapitulating the effect of STING agonism on CAFs observed *in vivo* (Fig. 6M and N). Conversely, the STING antagonist H151 resulted in a significant decrease in ifCAF signature scores (Supplementary Fig. S11B). Surprisingly, a significant decrease in myCAF signature scores was also observed in both monoculture and coculture (Supplementary Fig. S11C), indicating that endogenous STING activity is needed for the ifCAF phenotype but also for myCAF differentiation.

Furthermore, we observed that targets of CAF ligands in epithelial cells that were repressed by MSA-2 *in vivo* (Fig. 6K) were significantly lower in DMXAA treated tumor cells in coculture but not in monoculture (Fig. 6O). This demonstrates that DMXAA treated CAFs are directly modulating the expression of such genes in tumor cells, confirming the ligand–target interaction defined *in vivo*. Similar to the situation *in vivo,* (Fig. 6K), we observed a borderline significant (*P* = 0.056) reduction in the EMT signature score in cocultured tumor cells treated with DMXAA (Fig. 6P). No difference was observed in monocultured tumor cells, demonstrating that the effect of STING agonism on CAFs mediates the reduction in the EMT signature score. Similar effects were observed when myCAF formation was suppressed through STING inhibition with H151 (Supplementary Fig. S11D). Several EMT genes were repressed in cocultured tumor cells following STING agonism but not in monoculture (Supplementary Fig. S11E). This included *Spp1*, which was strongly repressed by DMXAA in cocultured tumor cells but unchanged in DMXAA treated monocultures, demonstrating that the effect of DMXAA on CAFs regulates *Spp1* expression in tumor cells (Fig. 6Q). *Spp1* expression was also strongly repressed in STING agonist–treated tumor cells *in vivo* (Fig. 6R). Importantly, STING agonists can induce an ifCAF phenotype and suppress a myCAF phenotype, resulting in the disruption of prometastatic interactions between CAFs and tumor cells.

## Discussion

**Figure 7.**
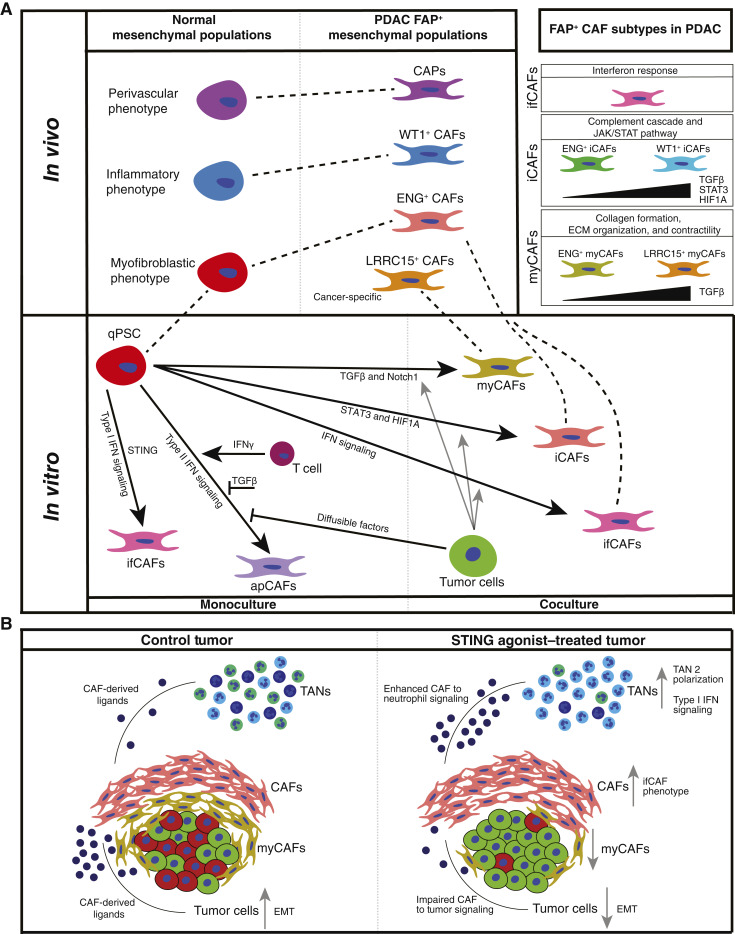
FAP^+^ mesenchymal cell heterogeneity in PDAC and regulation of CAF subtype formation. Schematic summary of findings. **A,** Top left, phenotypic similarities between mesenchymal subpopulations in the normal and PDAC stroma. Dotted lines show similar phenotypes as determined by integration. Top right, key phenotypic characteristics of iCAF, myCAF, and ifCAF subtypes. Bottom, summary of CAF subtype formation from PSCs *in vitro*. Arrows, differentiation paths with key regulators. Dotted lines depict similar phenotypes between *in vitro* and *in vivo* subpopulations as determined by integration. **B,** Effect of STING agonism on tumor cells, CAFs, and TANs.

In this study, we profiled FAP^+^ mesenchymal cells through scRNA-seq in human PDAC. We identified FAP^+^ CAFs and CAPs. Amongst CAFs, we defined CAF subpopulations, ENG^+^ CAFs, WT1^+^ CAFs, and LRRC15^+^ CAFs. Notably, we demonstrated that ENG^+^ CAFs, WT1^+^ CAFs, and CAPs shared phenotypic characteristics with mesenchymal populations in the healthy developing pancreas, whereas LRRC15^+^ CAFs represented a cancer-specific phenotype (Fig. 7A). In mouse models of PDAC, separate fibroblast lineages of the healthy pancreas can be distinguished through CD105 positivity and give rise to CAF subtypes in PDAC (48). CD105^-^ CAFs express *WT1, *which was also reported as a marker of fibroblast subtypes in the healthy pancreas and of mesothelial cells, which can form CAFs in PDAC (49, 51). Our data support such findings in a human context identifying CAF subtypes in PDAC expressing *ENG *(encoding CD105) and *WT1, *which correspond to mesenchymal populations in the healthy developing pancreas. Furthermore, mesenchymal subpopulations in the healthy human pancreas possess inflammatory and myofibroblastic features, reaffirming findings in mouse models of PDAC that mesenchymal populations of the healthy pancreas may be predisposed to inflammatory and myofibroblastic phenotypes (18).

We found that FAP^+^ CAFs are comprised primarily of iCAF and myCAF subtypes that can be separated based on expression of *LRRC15*, *WT1*, and *ENG*. LRRC15^+^ myCAFs can be distinguished from ENG^+^ myCAFs through enhanced TGFβ signaling, supporting previous findings that TGFβ drives LRRC15^+^ CAF formation (18, 65). WT1^+^ iCAFs differed from ENG^+^ iCAFs with strong engagement of pathways and transcription factors associated with hypoxia and inflammation but also elevated TGFβ signaling (Fig. 7A). A recent study demonstrated that *WT1 *expressing mesothelial cells can acquire a WT1^+^ CAF phenotype in response to IL1α and TGFβ treatment, which coincides with enhanced NFκB1, STAT3, SMAD3, and SMAD4 transcription factor activities observed in WT1^+^ CAFs in our data (49). Thus, WT1^+^ iCAFs may correspond to mesothelial-derived CAFs.

We also identified and validated the presence of a novel CAF subtype, ifCAFs, in human PDAC. In a mouse model of breast cancer, a subpopulation of CAFs characterized by an IFN response, interferon-licensed CAFs (ilCAF) have been described (53). Such ilCAFs shared an interferon-response phenotype observed in ifCAFs identified in this study. ilCAFs were induced following antibody blocking of TGFβ and were associated with a tumor-restraining effect through enhancing T-cell infiltration. However, ilCAFs were also associated with MHCII antigen presentation and may therefore represent apCAFs. An additional study profiling human breast cancer with scRNA-seq identified a population of CAFs with a type I interferon-response phenotype, which did not exhibit MHCII antigen presentation (66). These CAFs may correspond to ifCAFs identified in the present study, suggesting that this subtype is observed in different cancer types.

We were able to recapitulate CAF differentiation *in vitro* using a coculture model system comprised of KPC-derived tumor organoids and PSCs. PSCs are FAP-expressing cells characterized by the presence of cytoplasmic lipid–rich droplets in their quiescent state, which can form CAFs (4). We previously isolated FAP-expressing PSCs and validated their capacity to form cytoplasmic lipid droplets (4). We demonstrated that qPSCs cocultured with tumor cells can differentiate into myCAFs, iCAFs, and ifCAFs *in vitro*. Our scRNA-seq data at varying stages of differentiation in coculture with tumor cells demonstrated that PSCs undergo activation toward an inflammatory phenotype in early stages of coculture before later differentiating into myCAFs and ifCAFs or retaining an iCAF identity. We reaffirmed the role of TGFβ signaling in myCAF differentiation and identified Notch signaling as the potential unknown juxtacrine signaling component involved in their formation. Thus, signals derived from only tumor cells are sufficient to induce these CAF subtypes, and their differentiation can be modeled *in vitro*.

By integrating scRNA-seq data profiling qPSCs in monoculture with mesenchymal cells of the healthy pancreas (27), we found that PSCs correspond to a myofibroblastic subpopulation of mesenchymal cells. We have shown that this mesenchymal subpopulation corresponds to ENG^+^ CAFs identified in PDAC. Strengthening this relationship, we show that PSC-derived CAFs *in vitro* cluster together with ENG^+^ CAFs *in vivo*. In addition, we found that myCAFs formed from PSCs in coculture cluster together with LRRC15^+^ CAFs, suggesting PSCs may be the cellular origin of this population. In support of this, a previous study found that an Eng^+^ myofibroblastic subpopulation of fibroblasts gave rise to Lrrc15^+^ CAFs in a mouse model of PDAC(18). Together, we demonstrate that PSCs represent a subpopulation of mesenchymal cells with a myofibroblastic phenotype and that PSC-derived CAFs formed *in vitro* correspond to ENG^+^ and LRRC15^+^ CAFs but not WT1^+^ CAFs or CAPs. This is in accordance with previous findings that PSC-derived CAFs represent a subpopulation of all CAFs, although PSC-derived CAFs were shown to be less abundant than those found in our data (67). Together, our analysis elucidates the broad subtypes of mesenchymal cells found in PDAC and provides insight into the differentiation of specific subtypes in response to tumor-derived signals.

We elucidated that activated T cells, through type II IFN signaling, can stimulate MHCII antigen presentation in PSCs. apCAFs have been described in PDAC (17) but were not detected in our FAP^+^ cells from human PDAC. apCAFs have been proposed to originate from mesothelial cells and may therefore not express FAP (49). In addition, we corroborated previous findings that tumor cell–derived signals could suppress MHCII antigen presentation, which can also account for the absence of FAP^+^ apCAFs detected *in vivo* (48).

We demonstrated that the ifCAF phenotype can be induced with STING agonists, which promoted a type I IFN response without inducing MHCII presentation. We provide novel insights into the influence of administering STING agonists on CAFs *in vivo*, finding that rather than inducing an increase in the proportion of the ifCAF subtype, a global IFN response is induced amongst CAFs alongside a decrease in the prevalence of myCAFs. In addition to the effect on CAFs, we found that STING agonism suppressed tumor cell invasiveness and proliferation and induced a shift toward antitumor immunity in TANs. STING agonists have been shown to promote antitumor immunity in mouse models of PDAC, though previous studies have focused mainly on the drugs effect on macrophages and T cells (60, 61). Recent studies have highlighted the importance of TANs in modulating disease progression in PDAC, and type I IFN signaling is associated with imposing a tumor-restraining phenotype on TANs (62–64). We demonstrated that STING agonists drastically modulate TAN polarization and ifCAFs directly promote an antitumor phenotype in TANs. TAN polarization by STING agonism observed *in vivo* may partly be a direct consequence of the drug, but we find that ifCAF-derived signals promoted the same effect *in vitro*, suggesting that ifCAFs synergize with the drug to augment its effects and may therefore be critical targets for inducing antitumor immunity.

We found that ligands suppressed in CAFs by STING agonists were highly expressed in myCAFs. The targets of such ligands in tumor cells related primarily to EMT. We subsequently demonstrated that treating *in vitro* cocultures with STING agonists promoted ifCAF formation and disrupted formation of myCAFs, suppressing CAF-mediated induction of EMT genes in tumor cells (Fig. 7B). We also found that STING antagonists could repress myCAF formation. It has been demonstrated that STING antagonists can inhibit myCAF formation in ovarian cancer (68). Inhibiting myCAF formation either with STING agonists or antagonists was associated with a reduction in EMT genes in tumor cells, directly evidencing myCAFs as a prometastatic CAF subpopulation. Despite myCAFs being implicated in restraining tumor growth (14), studies have suggested they may also promote EMT, and our data supports such a role (69, 70). We find several EMT-related genes whose induction in tumor cells by CAFs is suppressed by STING agonism, including *SPP1*. CAFs have been shown to promote *SPP1 *expression in tumor cells, which promotes invasiveness (71). It has been demonstrated in mouse models of PDAC that tumor cells with elevated IFN signaling can induce an interferon-response phenotype globally in CAFs (72). In contrast to our findings, the authors showed that CAFs with high IFN signaling promote tumor growth. This suggests that tumors could respond variably to induction of an IFN response in CAFs and warrants future investigation.

Our results provide novel insights into mechanisms through which CAFs help orchestrate the antitumor properties of STING agonists. Although STING agonists have shown promising tumor-restraining properties in several cancer types (73), the physiologic and chemical properties of STING agonists, in addition to the dangers of their systemic administration, have limited their efficacy in clinical trials (74). Our findings provide the exciting opportunity that specifically targeting CAFs with STING agonists can promote an antitumor phenotype in TANs in addition to suppressing the prometastatic influence of CAFs on tumor cells in PDAC.

## Supplementary Material

Supplementary Data Supplementary data figure legends and references

Table S1 Clinical Characteristics of human PDAC samples

Table S2 Cell metadata, differentially expressed genes, gene-set enrichment analysis results and differentially active transcription factors for mesenchymal subtypes characterized in Figure 1.

Table S3 Published gene signatures of mesenchymal cell types.

Table S4 Cell annotation and metadata of previously published scRNAseq data from Olaniru et al, 2023.

Table S5 Cell metadata, differentially expressed genes, gene-set enrichment analysis results and differentially active transcription factors for CAF subtypes characterized in Figure 2.

Table S6 Cell metadata, differentially expressed genes, gene-set enrichment analysis results and differentially active transcription factors for PSC subtypes in monoculture and co-cultures characterized in Figure 3.

Table S7 Human and mouse orthology table.

Table S8 Cell metadata, differentially expressed genes, gene-set enrichment analysis results and differentially active transcription factors for PSC subtypes in time-course characterized in Figure 4.

Table S9 Differentially expressed genes and gene-set enrichment analysis results for PSCs treated with DMXAA, IFNγ or Activated T-cells in Figure S6.

Table S10 Cell metadata, differentially expressed genes and MSA-2 regulated ligands in CAFs and targets in neutrophils and epithelial cells for scRNAseq data profiling orthotopically transplanted tumors in Figure 5 & 6.

Table S11 Differentially expressed genes in neutrophils exposed to PSC conditioned media and PSC and tumor cell monocultures and co-cultures treated with DMXAA and H151 in Figure 6, S10 and S11.

Table S12 PCR primers sequences.

Figure S1 FAP+ mesenchymal subtypes populate the PDAC stroma.

Figure S2 alidation of FAP+ CAF subpopulations in human PDAC.

Figure S3 FAP+ mesenchymal subtypes reflect mesenchymal heterogeneity present in the healthy developing pancreas.

Figure S4 Characterization of FAP+ iCAF and myCAF subtypes in PDAC.

Figure S5 Characterization of FAP+ CAF subtypes in PDAC.

Figure S6 An in vitro murine co-culture model system can recapitulate CAF heterogeneity observed in vivo.

Figure S7 A time-course profiling CAF differentiation in vitro through scRNAseq elucidates the dynamics of CAF subtype formation.

Figure S8 Type I and type II interferon pathways govern interferon-response CAF (ifCAF) and antigen-presenting CAF formation.

Figure S9 STING agonism reprograms the tumor microenvironment and suppresses metastasis in a mouse model of PDAC

Figure S10 STING agonism in CAFs promotes TAN polarization.

Figure S11 STING modulation in CAFs directs subtype formation which disrupts pro-metastatic influences of CAFs on tumor cells.
