# Supplementary material for: Dissecting FAP+ Cell Diversity in Pancreatic Cancer Uncovers an Interferon-Response Subtype of Cancer-Associated Fibroblasts with Tumor-Restraining Properties
Source: Cancer Res. 2025 Apr 11;85(13):2388–411. doi: 10.1158/0008-5472.CAN-23-3252 (PMC12214878; doi:10.1158/0008-5472.CAN-23-3252)
Supplement: Supplementary Data — figure legends and references [file can-23-3252_supplementary_data_suppsd.docx]

**Supplemental Data**

Dissecting FAP+ Cell Diversity in Pancreatic Cancer Uncovers an Interferon-Response Subtype of Cancer-Associated Fibroblasts with Tumor-Restraining Properties

**Supplemental Figures:**

**Figure S1.** FAP^+^ mesenchymal subtypes populate the PDAC stroma.

**Figure S2.** Validation of FAP+ CAF subpopulations in human PDAC.

**Figure S3.** FAP^+^ mesenchymal subtypes reflect mesenchymal heterogeneity present in the healthy developing pancreas.

**Figure S4.** Characterization of FAP^+^ iCAF and myCAF subtypes in PDAC.

**Figure S5.** Characterization of FAP^+^ CAF subtypes in PDAC.

**Figure S6.** An *in vitro* murine co-culture model system can recapitulate CAF heterogeneity observed *in vivo*.

**Figure S7.** A time-course profiling CAF differentiation *in vitro* through scRNAseq elucidates the dynamics of CAF subtype formation.

**Figure S8.** Type I and type II interferon pathways govern interferon-response CAF (ifCAF) and antigen-presenting CAF formation.

**Figure S9.** STING agonism reprograms the tumor microenvironment and suppresses metastasis in a mouse model of PDAC

**Figure S10.** STING agonism in CAFs promotes TAN polarization.

**Figure S11.** STING modulation in CAFs directs subtype formation which disrupts pro-metastatic influences of CAFs on tumor cells.

**Supplemental Tables:**

**Table S1.** Clinical characteristic of 5 human PDAC samples.

**Table S2.** Cell metadata, differentially expressed genes, gene-set enrichment analysis results and differentially active transcription factors for mesenchymal subtypes characterized in Figure 1.

**Table S3.** Published gene signatures of mesenchymal cell types.

**Table S4.** Cell annotation and metadata of previously published scRNAseq data from Olaniru *et al*, 2023.

**Table S5.** Cell metadata, differentially expressed genes, gene-set enrichment analysis results and differentially active transcription factors for CAF subtypes characterized in Figure 2.

**Table S6.** Cell metadata, differentially expressed genes, gene-set enrichment analysis results and differentially active transcription factors for PSC subtypes in monoculture and co-cultures characterized in Figure 3.

**Table S7.** Human and mouse orthology table.

**Table S8.** Cell metadata, differentially expressed genes, gene-set enrichment analysis results and differentially active transcription factors for PSC subtypes in time-course characterized in Figure 4.

**Table S9.** Differentially expressed genes and gene-set enrichment analysis results for PSCs treated with DMXAA, IFNγ or Activated T-cells in Figure S6.

**Table S10.** Cell metadata, differentially expressed genes and MSA-2 regulated ligands in CAFs and targets in neutrophils and epithelial cells for scRNAseq data profiling orthotopically transplanted tumors in Figure 5 & 6.

**Table S11.** Differentially expressed genes in neutrophils exposed to PSC conditioned media and PSC and tumor cell monocultures and co-cultures treated with DMXAA and H151 in Figure 6, S10 and S11.

**Table S12.** PCR primers sequences.

**Supplementary Figure Legends**

**Figure S1. FAP^+^ mesenchymal subtypes populate the PDAC stroma. (A-B)** Quantification of sample contribution to each cluster displayed as UMAP plots (A) and bar plots (B). **(C)** Cluster tree depicting cluster relationships at different resolutions. Dots represent clusters and are colored by resolution and sized by number of cells within cluster. Lines are colored by number of cells separating from clusters at increased resolutions. **(D)** Heatmap showing hierarchical clustering and scaled expression levels of differentially expressed genes between mesenchymal cell subtypes. **(E-F)** Scatter plots depicting mean normalized counts for gene signatures from Wang *et al*^1^ (E) and Wu *et al*^2^ (F) in clusters. Each point represents a cluster.

**Figure S2. Validation of FAP^+^ CAF subpopulations in human PDAC. (A)** H&E staining of 5 human PDAC samples profiled with scRNAseq. Pathologist’s annotation of tumor ECM as dense, moderate or loose above images. **(B)** Representative immunofluorescence (IF) co-staining of FAP and markers of CAF subpopulations (red) in one human PDAC sample (Sample 3, S3). Counterstain, DAPI (blue). (Bottom) Higher magnification illustrating co-staining in cells. The colors of markers are indicated above each image. **(C)** Unsupervised clustering of mesenchymal cells derived from scRNAseq dataset profiling PDAC from Peng *et al*^4^ integrated with rPCA and visualized through UMAP. **(D)** UMAP plots showing Z-scores for gene signatures of CAF subpopulations defined in Figure 1.

**Figure S3. FAP^+^ mesenchymal subtypes reflect mesenchymal heterogeneity present in the healthy developing pancreas. (A)** Stacked violin plots showing normalized gene expression levels of differentially expressed gene markers of mesenchymal cell subtypes in the healthy normal pancreas from Olaniru *et al*^3^. **(B)** Gene-set enrichment analysis showing enriched HALLMARK, KEGG and Reactome pathways between mesenchymal cell subtypes identified in the healthy developing pancreas^3^. Size of dots represents fold-change in pathway enrichment and color displays q values of pathways. (**C-D)** Stacked violin plots showing normalized gene expression levels of differentially expressed gene markers of mesenchymal cell subtypes in the healthy normal pancreas from Olaniru *et al*^3^. **(F)** Unsupervised clustering of mesenchymal cells derived from the normal developing pancreas from Olaniru *et al*^3^ and PDAC (Figure 1B) integrated with rPCA and visualized through UMAP. **(G-H)** Quantification of disease state contribution to each cluster displayed as UMAP plots (G) and bar plots (H). **(I-J)** Quantification of mesenchymal subpopulations contribution to each cluster displayed as UMAP plots (I) and bar plots (J). **(K)** Heatmap of scaled expression levels for differentially expressed gene markers of corresponding mesenchymal clusters identified in the healthy developing pancreas and PDAC. Genes were selected as differentially expressed in both normal pancreas and PDAC. **(L)** GSEA showing enriched HALLMARK, KEGG and Reactome pathways in corresponding mesenchymal clusters identified in the healthy developing pancreas and PDAC. Size of dots represents fold-change in pathway enrichment and color displays q values of pathways. **(M)** Heatmap of scaled activities for differentially active transcription factors of corresponding mesenchymal clusters identified in the healthy developing pancreas and PDAC. **(N)** Schematic illustration of corresponding mesenchymal subpopulations in the normal pancreas and PDAC. Dotted lines depict associations determined through integration.

**Figure S4. Characterization of FAP^+^ iCAF and myCAF subtypes in PDAC. (A-B)** Quantification of sample contribution to each cluster displayed as UMAP plots (A) and bar plots (B). **(C)** Scatter plots depicting mean gene signature Z-scores for two inflammatory pathways in clusters. Each point represents a cluster. **(D-F)** Scatter plots depicting mean gene signature Z-scores for iCAFs and meCAFs (D), iCAFs (E) and myCAFs (F) from Wu *et al*^1^ and Elyada *et al*^5^ in clusters. Each point represents a cluster. **(G-H)** Proportion of iCAF, myCAF and other CAF subtypes in (G) tumor samples and (H) between tumors samples with dense and moderate/loose desmoplasia. **(I)** Heatmap of scaled activity scores for differentially active transcription factor activities in iCAF and myCAF subtypes. **(J)** Heatmap of scaled expression levels for differentially expressed genes in iCAF and myCAF subtypes. **(K)** GSEA showing enriched HALLMARK, KEGG and Reactome pathways in iCAF and myCAF subtypes. Selected pathways visualized in a dot plot, size and color of dots represents fold-change in pathway enrichment and s q values, respectively.

**Figure S5. Characterization of FAP^+^ CAF subtypes in PDAC. (A-D)** Stacked violin plots showing normalized gene expression levels of VEGFA^+^ CAFs (A), WT1^+^ CAF (B), PI16^+^ CAFs (C) and CAP-like CAFs (D) markers. **(E-G)** (E) Unsupervised clustering of CAFs from Peng *et al*^4^ visualized through UMAP, (F) violin plots show *FAP* expression in CAF clusters, (G) violin plots showing CAF subtype signature Z-scores from Figure 2 in CAF clusters.

**Figure S6. Characterization of FAP^+^ CAF subtypes in PDAC. An *in vitro* murine co-culture model system can recapitulate CAF heterogeneity observed *in vivo*. (A)** Representative brightfield image of co-culture taken at day 4 of co-culture. **(B-C)** Quantification of biological replicate contribution to each cluster displayed as UMAP plots (A) and bar plots (B). **(D)** UMAP displaying culture condition. **(E)** GSEA showing enriched HALLMARK, KEGG and Reactome pathways in PSC and CAF subtypes. Selected pathways visualized in a dot plot, size of dots represents fold-change in pathway enrichment and color displays q values of pathways. **(F)** Heatmap of scaled activity scores for differentially active transcription factors in PSC and CAF subtypes. **(G-J)** Violin plots showing gene signature Z-scores for iCAFs (G), ifCAFs (H), myCAFs (I) and Crabp1^+^ CAFs (J) from Figure 3B.

**Figure S7. A time-course profiling CAF differentiation *in vitro* through scRNAseq elucidates the dynamics of CAF subtype formation. (A)** Unsupervised clustering of FACS sorted single-cells for 15 cDNA libraries from co-cultures at 7 time-points (PSC5 + mT3) visualized through UMAP. **(B-C)** Quantification of time-point contribution to each cluster displayed as UMAP plots (B) and bar plots (C). **(D)** UMAP plot (B) showing trajectory of CAF differentiation in co-culture inferred by Slingshot. **(E)** UMAP plot (B) split by lineage and colored by pseudotime inferred by Slingshot. **(F)** Scatter plots showing cell pseudotime values across time-points for each lineage. **(G)** Gene-set enrichment analysis showing enriched HALLMARK, KEGG and Reactome pathways between PSCs at day 0 and day 1 of co-culture. Size of dots represents fold-change in pathway enrichment and color displays q values of pathways. **(H-I)** Quantification of technical replicate contribution to each cluster displayed as UMAP plots (H) and bar plots (I). **(J)** Violin (top) and UMAP (bottom) plots depicting Z-Scores of gene signatures of Crabp1^+^ CAFs from Figure 3B.

**Figure S8. Type I and type II interferon pathways govern interferon-response CAF (ifCAF) and antigen-presenting CAF (apCAF) formation. (A)** Bar plots showing qPCR quantification of ifCAF gene markers expression following treatment of monocultured PSCs with 1µM, 10µM and 100µM of DMXAA for 6 hours. Data is shown as fold change relative to vehicle control. Error bars show standard deviation. **(B)** Quantification of CXCL10 protein levels through ELISA in monocultured PSCs treated with vehicle (DMSO) or DMXAA (100µM) after 6 hours. **(C-H)** Quantification of Z-scores for ifCAF (C) iCAF (D), myCAF (E), qPSC 1 (F), qPSC2 (G) and Crabp1^+^ CAF (H) gene signatures (Figure 3B) in vehicle or DMXAA (100µM) treated PSCs in monoculture or co-culture with tumor cells. PSC4 ± mT4 (n=2) and PSC5 ± mT3 (n=2). **(I-K)** Proportion of MHCII^+^ PSCs in monoculture or co-culture with tumor cells determined by flow cytometry following stimulation with (I) recombinant IFNγ (10ηg/ml) at day 4, (J) recombinant IFNγ (10ηg/ml) at day 0 or (K) activated T-cells at day 4. **(L)** Proportion of MHCII^+^ PSCs in monoculture determined by flow cytometry following stimulation with recombinant IFNγ in control media, in combination with recombinant TGFβ or in tumor cell conditioned media. **(M)** Proportion of MHCII^+^ PSCs in monoculture determined by flow cytometry following stimulation with recombinant IFNγ (10ηg/ml) or STING agonist DMXAA (100µM). **(N)** Gene-set enrichment analysis showing enriched HALLMARK, KEGG and Reactome pathways between MHCII^+^ PSCs following stimulation with recombinant IFNγ (10ηg/ml) or PSCs stimulated with DMXAA (100µM) against relevant control PSCs. Selected pathways visualized in a dot plot, size of dots represent number of differentially expressed genes present in pathways and color displays adjusted p value of pathways. **(O)** Z-values for ISMARA calculated transcription factor activity scores for MHCII^+^ PSCs following stimulation with recombinant IFNγ (10ηg/ml) or PSCs treated with DMXAA (100µM) against relevant control PSCs. All panels n=4 (2 biological replicates and 2 technical replicates. Samples are compared through Mann-Whitney test (B and J) and Multiple t tests (C-H), 2-way ANOVA with Tukey’s multiple comparison test (I, K and L) and Kruskal-Wallis test with Dunn’s multiple comparison test (M). ns, not significant; *p < 0.05, **p < 0.01, ***p < 0.001, ****p < 0.0001.

**Figure S9. STING agonism reprograms the tumor microenvironment and suppresses metastasis in a mouse model of PDAC.** scRNAseq profiling of orthotopically transplanted tumors. **(A)** Dot plot showing canonical markers of cell types in clusters. **(B-C)** Violin plots of Z-scores in CAF subtypes for gene signatures of (B) CAF subtypes identified *in vitro* in Figure 3 and (C) FAP^+^ CAF subtypes defined in human PDAC *in vivo* Figure 2. **(D)** UMAP plot showing distribution of cells in control and MSA-2 treated tumors. **(E)** Violin plots showing Z-scores for the HALLMARK Interferon Alpha Response gene signature in TAN clusters. **(F)** Boxplots showing Z-scores of gene signatures for (left) CD8 Activation and (right) Cytolytic effect from Azizi *et al*^6^, in T-cells. Compared through Wilcoxon rank-sum test. ns, not significant; *p < 0.05, **p < 0.01, ***p < 0.001, ****p < 0.0001.

**Figure S10. STING agonism in CAFs promotes TAN polarization and suppresses their pro-metastatic influence on tumor cells. (A)** Box plots showing Z-scores of MSA-2 induced ligand expression in CAFs between CAF subtypes in orthotopically transplanted tumors. **(B)** Representative FACS plots showing isolation of neutrophils (CD45^+^ Ly6G^+^ CD11b^+^) from bone-marrow. **(C-D)** Bone-marrow derived primary neutrophils exposed to conditioned media from PSCs treated with DMSO or DMXAA (100µM) (Figure 6E). (C) Volcano plot showing differentially expressed genes. (D) Z-Scores for gene signatures of TAN 1 and TAN3 subtypes. Points represent paired replicates, conditioned media derived from PSC4 (n=3) and PSC5 (n=3). Samples are compared through paired wilcoxon rank sum test (D). ns, not significant; *p < 0.05, **p < 0.01, ***p < 0.001, ****p < 0.0001.

**Figure S11. STING antagonism in CAFs suppresses ifCAF and myCAF formation. (A)** Percentage of tumor cell covered area for monocultured tumor cells mT3 (n=3) & mT4 (n=3) and tumor cells co-cultured with PSCS mT3 + PSC5 (n=3) & mT4 + PSC4 (n=3) over a time-course treated with vehicle control, DMXAA (100µM), Gemcitabine (5nM) or DMXAA + Gemcitabine. **(B-D)** DMSO (0.2%) and H151 (100µM) added to PSC and tumor cell monocultures and co-cultures before FACS isolation of cell types and bulk RNAseq. Z-scores for gene signature expression in PSCs (B & C) and tumor cells (D). ifCAF (B) and myCAF (C) signatures from Figure 3, (D) HALLMARK EMT signature. Points represent paired replicates, monocultures PSC4 (n=3), PSC5 (n=3), mT3 (n=3), mT4 (n=3) and co-cultures PSC4 + mT4 (n=3) and PSC5 + mT3 (n=3). (E) Box plots showing normalized expression values of EMT related genes in tumor cells in monoculture and co-culture in DMSO and DMXAA treated conditions. Samples are compared through 2way ANOVA with Dunnett’s multiple comparison test (A), paired wilcoxon rank sum test (B-D) and the Wald test from the DESeq2 package (D). ns, not significant; *p < 0.05, **p < 0.01, ***p < 0.001, ****p < 0.0001.

**References**

1. Wang Y, Liang Y, Xu H, Zhang X, Mao T, Cui J, et al. Single-cell analysis of pancreatic ductal adenocarcinoma identifies a novel fibroblast subtype associated with poor prognosis but better immunotherapy response. *Cell Discov*. **2021**;7.

2. Wu SZ, Al-Eryani G, Roden DL, Junankar S, Harvey K, Andersson A, et al. A single-cell and spatially resolved atlas of human breast cancers. *Nat Genet*. **2021**;53:1334–47.

3. Olaniru OE, Kadolsky U, Kannambath S, Vaikkinen H, Fung K, Dhami P, et al. Single-cell transcriptomic and spatial landscapes of the developing human pancreas. *Cell Metab*. **2023**;35:184-199.e5.

4. Peng J, Sun BF, Chen CY, Zhou JY, Chen YS, Chen H, et al. Single-cell RNA-seq highlights intra-tumoral heterogeneity and malignant progression in pancreatic ductal adenocarcinoma. *Cell Res*. **2019**;29:725–38.

5. Elyada E, Bolisetty M, Laise P, Flynn WF, Courtois ET, Burkhart RA, et al. Cross-species single-cell analysis of pancreatic ductal adenocarcinoma reveals antigen-presenting cancer-associated fibroblasts. *Cancer Discov*. **2019**;9:1102–23.

6. Azizi E, Carr AJ, Plitas G, Cornish AE, Konopacki C, Prabhakaran S, et al. Single-Cell Map of Diverse Immune Phenotypes in the Breast Tumor Microenvironment. *Cell*. **2018**;174:1293-1308.e36.
