# Supplementary figures and images for: Dissecting FAP+ Cell Diversity in Pancreatic Cancer Uncovers an Interferon-Response Subtype of Cancer-Associated Fibroblasts with Tumor-Restraining Properties
Source: Cancer Res. 2025 Apr 11;85(13):2388–411. doi: 10.1158/0008-5472.CAN-23-3252 (PMC12214878; doi:10.1158/0008-5472.CAN-23-3252)

Figure S1.

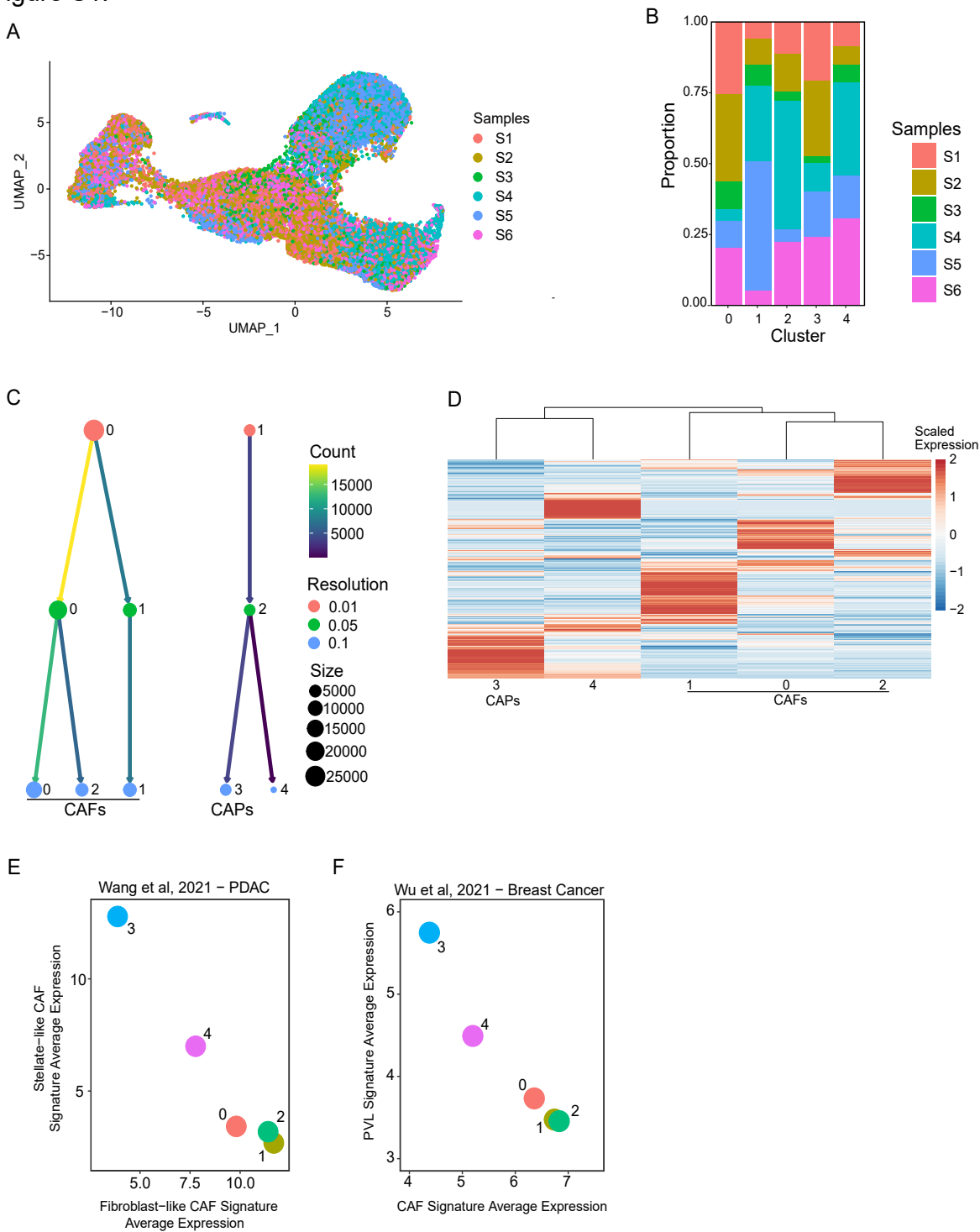

Supplement: Figure S1 — FAP+ mesenchymal subtypes populate the PDAC stroma. [file can-23-3252_figure_s1_suppsf1.pdf]

Figure S2.

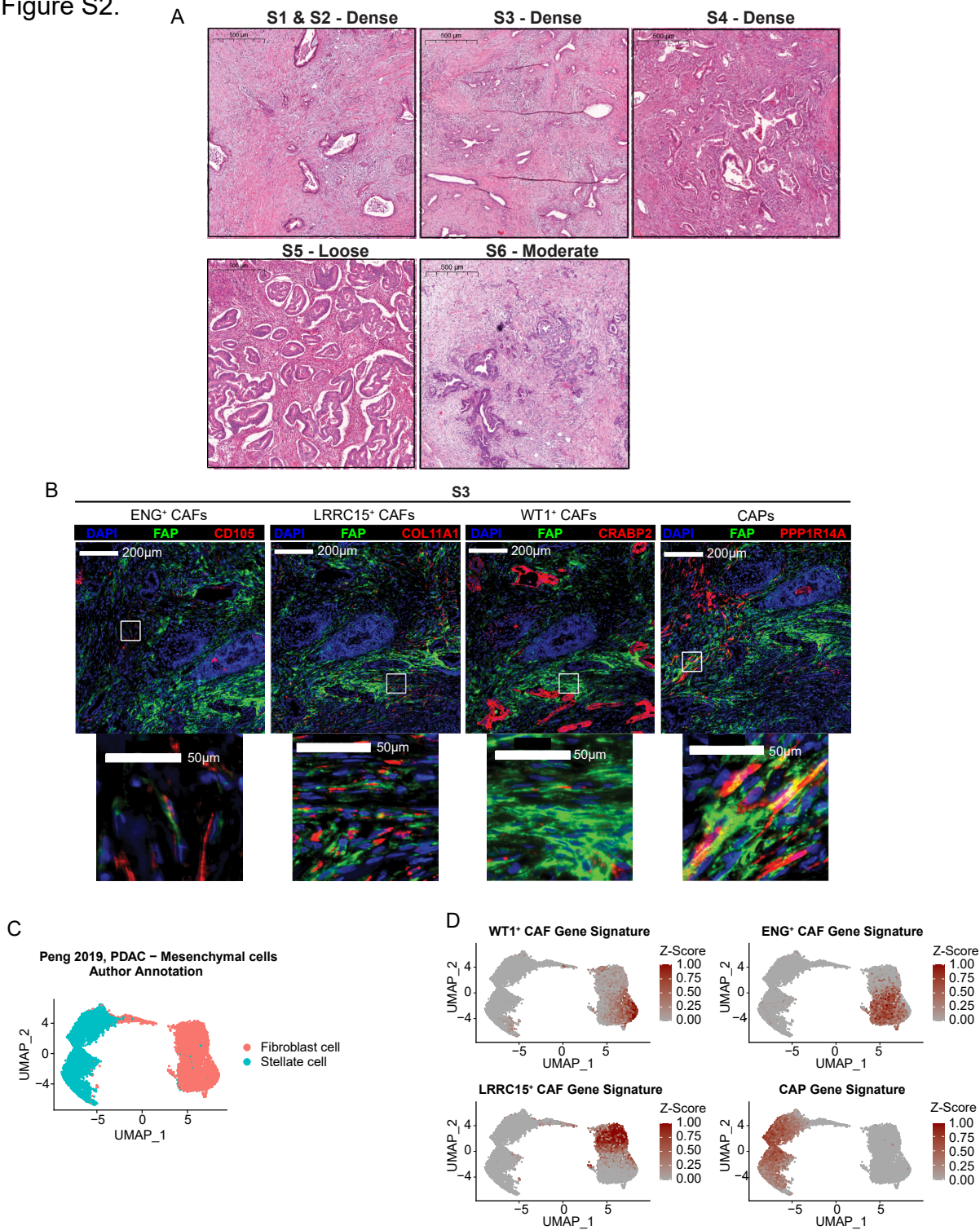

Supplement: Figure S2 — alidation of FAP+ CAF subpopulations in human PDAC. [file can-23-3252_figure_s2_suppsf2.pdf]

Figure S3.

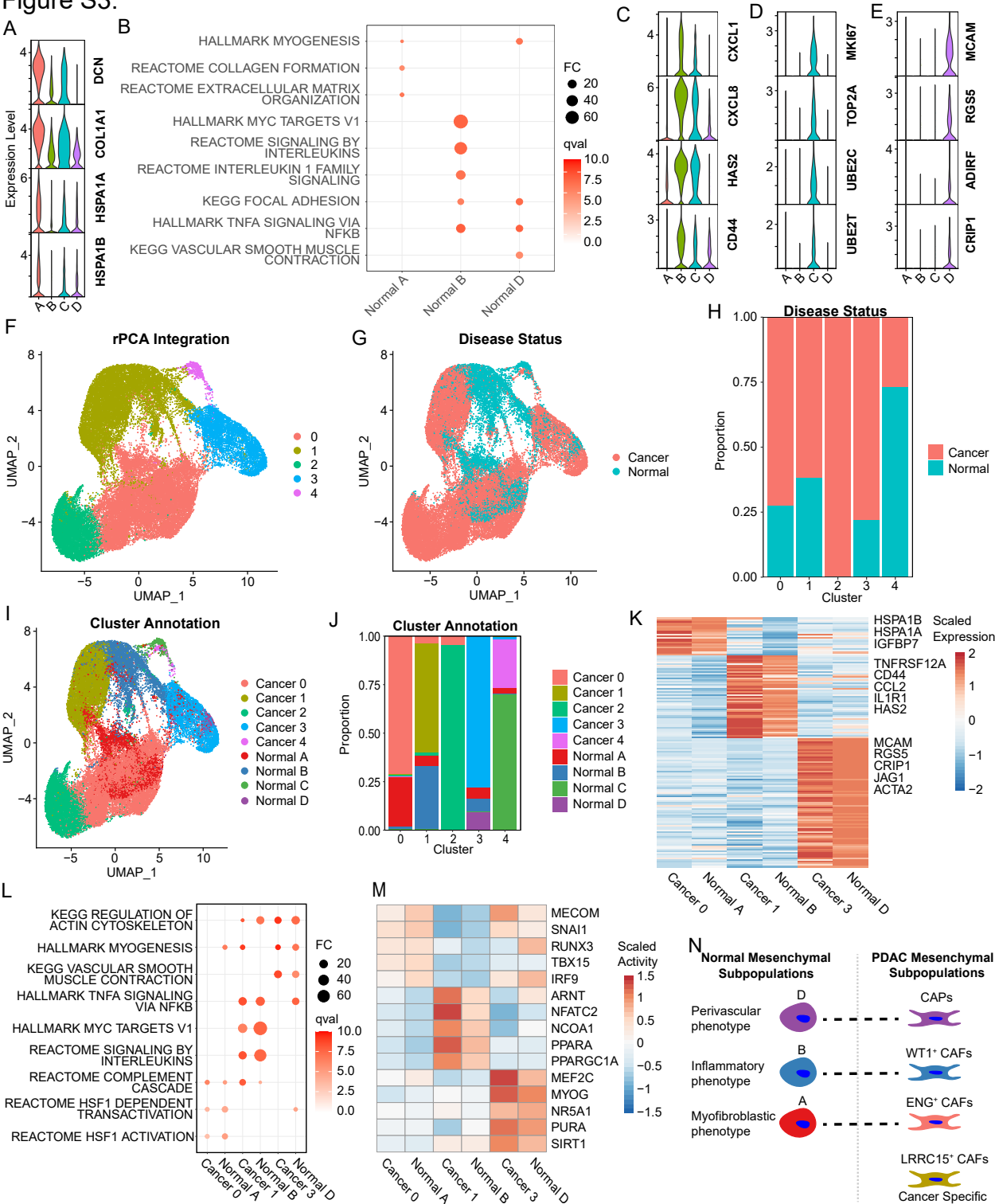

Supplement: Figure S3 — FAP+ mesenchymal subtypes reflect mesenchymal heterogeneity present in the healthy developing pancreas. [file can-23-3252_figure_s3_suppsf3.pdf]

Figure S4.

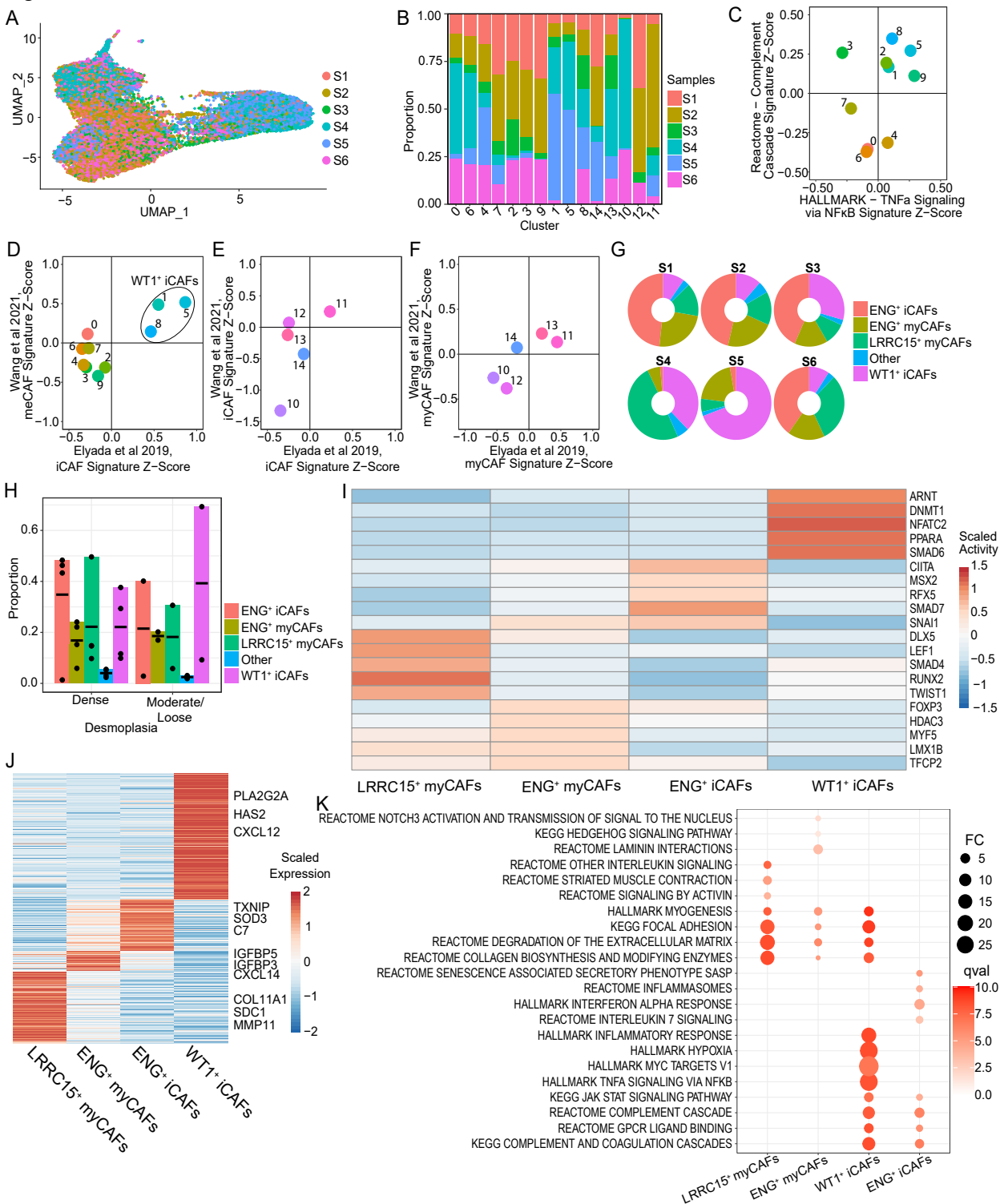

Supplement: Figure S4 — Characterization of FAP+ iCAF and myCAF subtypes in PDAC. [file can-23-3252_figure_s4_suppsf4.pdf]

Figure S5.

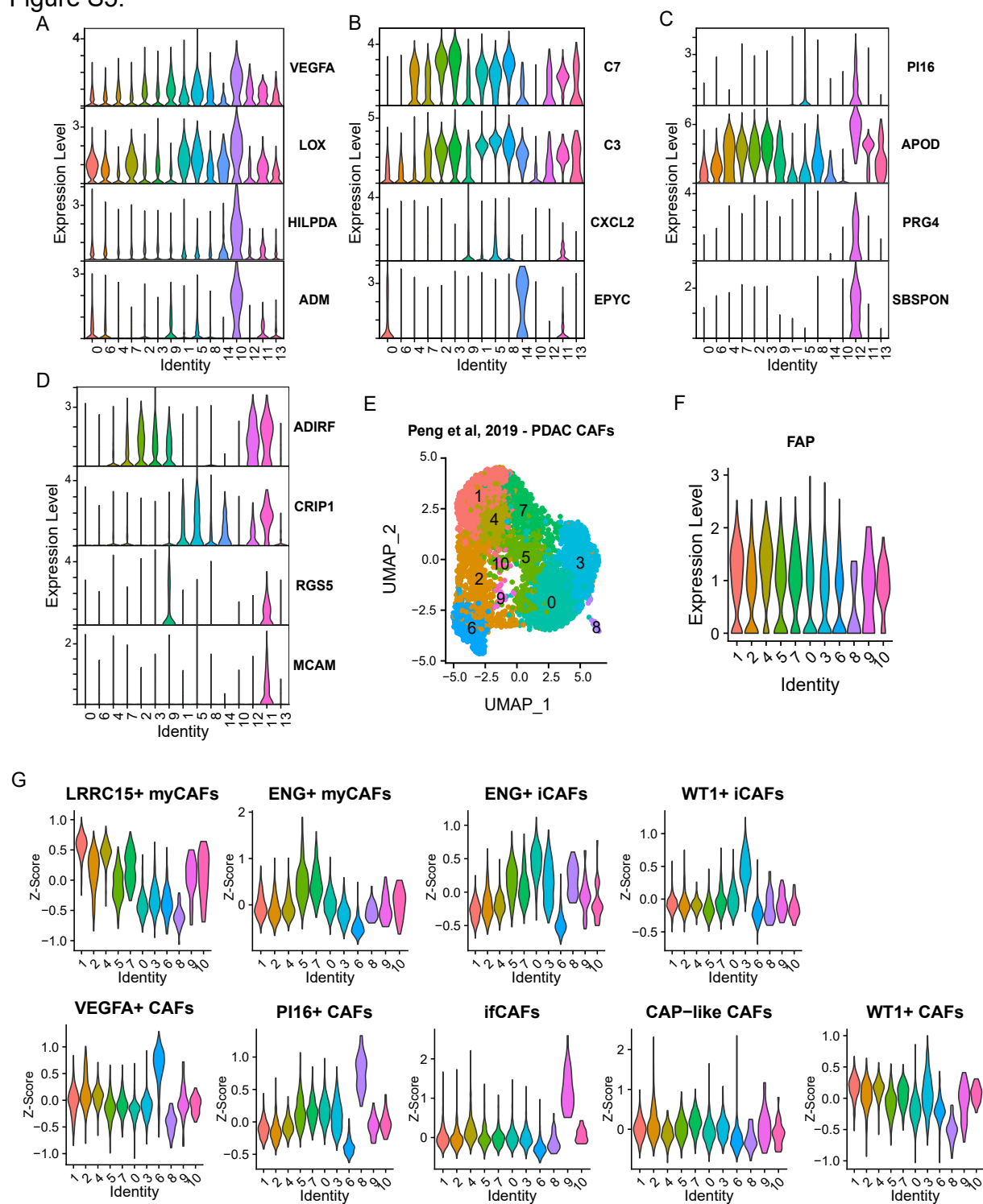

Supplement: Figure S5 — Characterization of FAP+ CAF subtypes in PDAC. [file can-23-3252_figure_s5_suppsf5.pdf]

Figure S6.

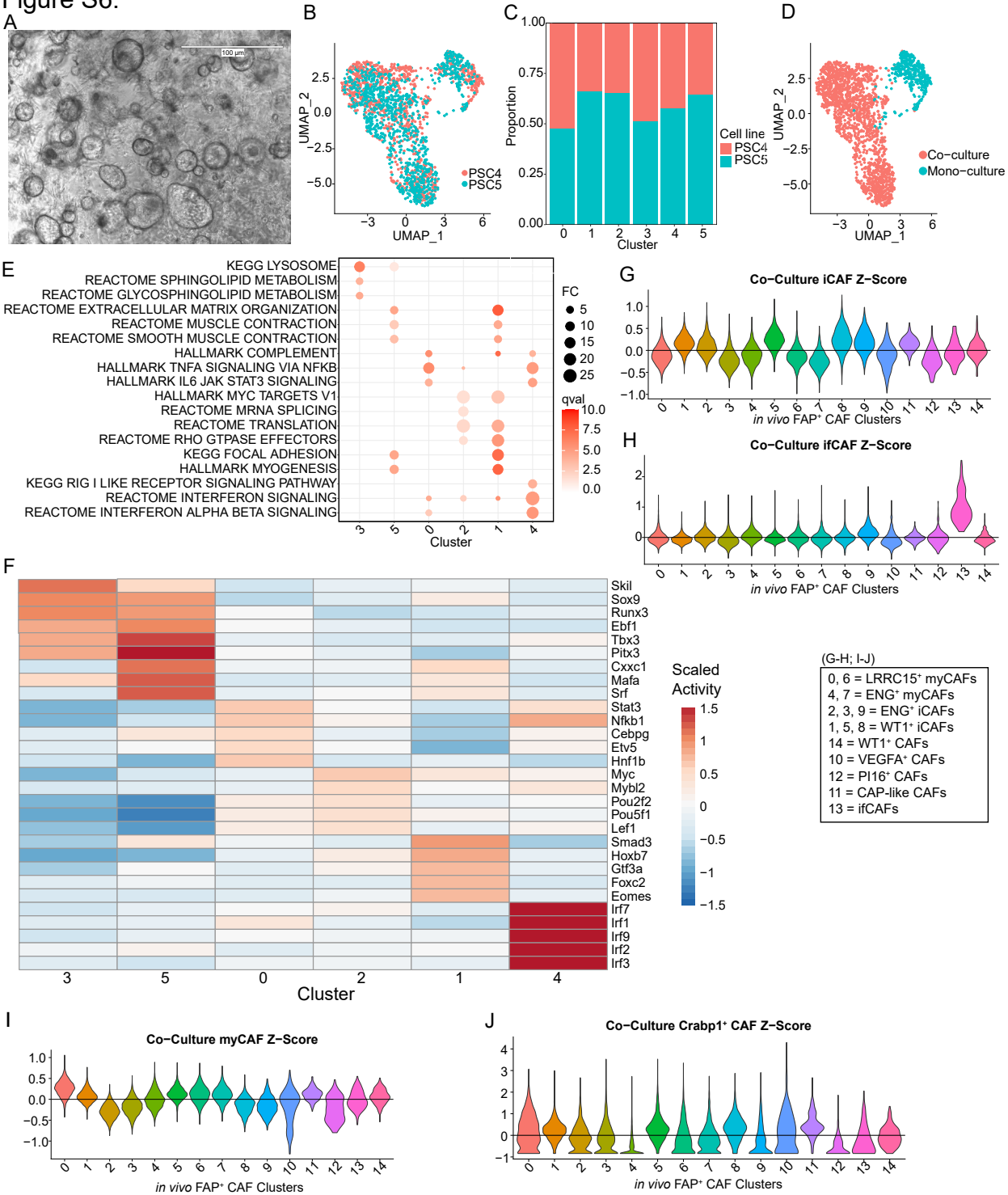

Supplement: Figure S6 — An in vitro murine co-culture model system can recapitulate CAF heterogeneity observed in vivo. [file can-23-3252_figure_s6_suppsf6.pdf]

Figure S7.

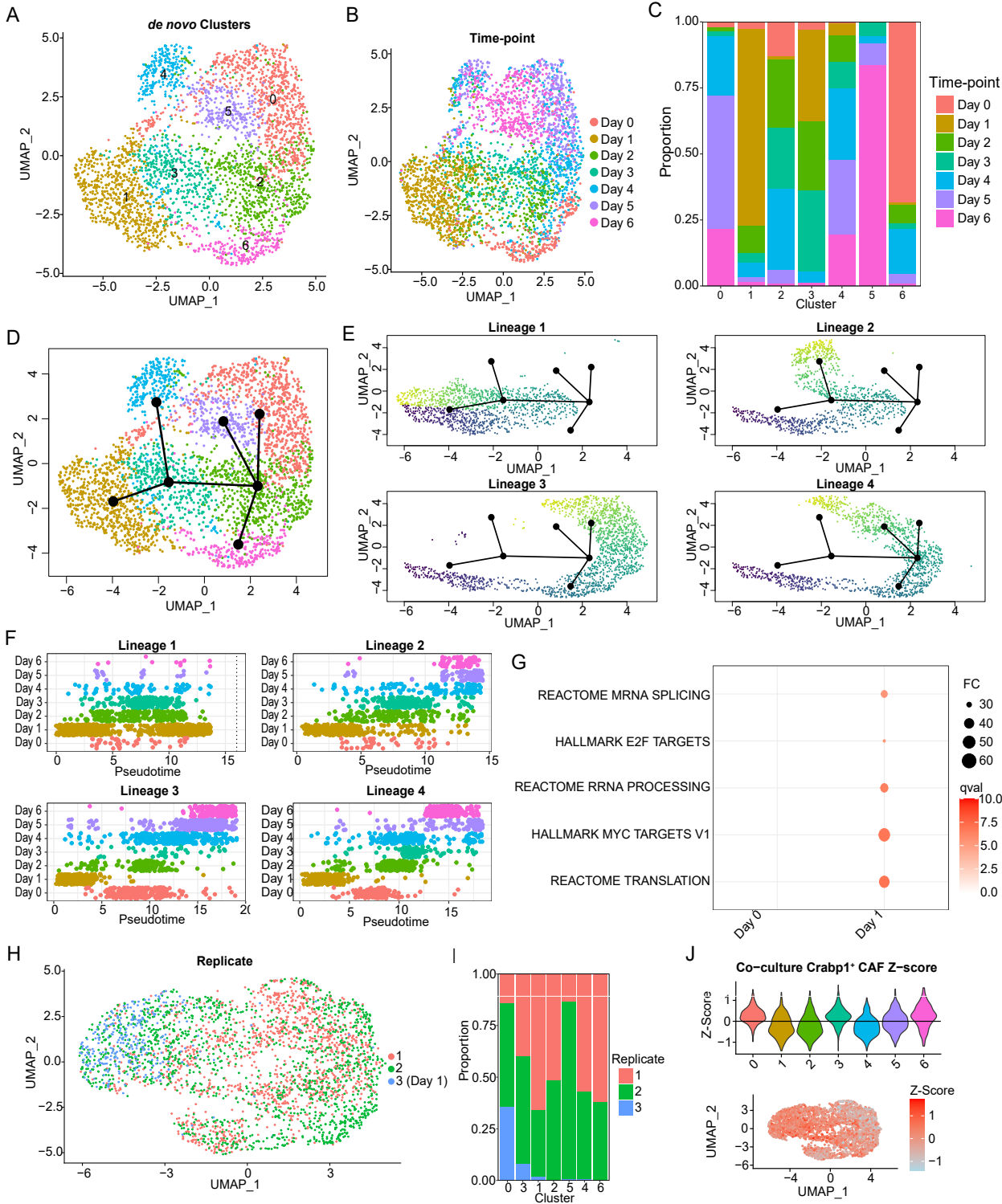

Supplement: Figure S7 — A time-course profiling CAF differentiation in vitro through scRNAseq elucidates the dynamics of CAF subtype formation. [file can-23-3252_figure_s7_suppsf7.pdf]

Figure S8.

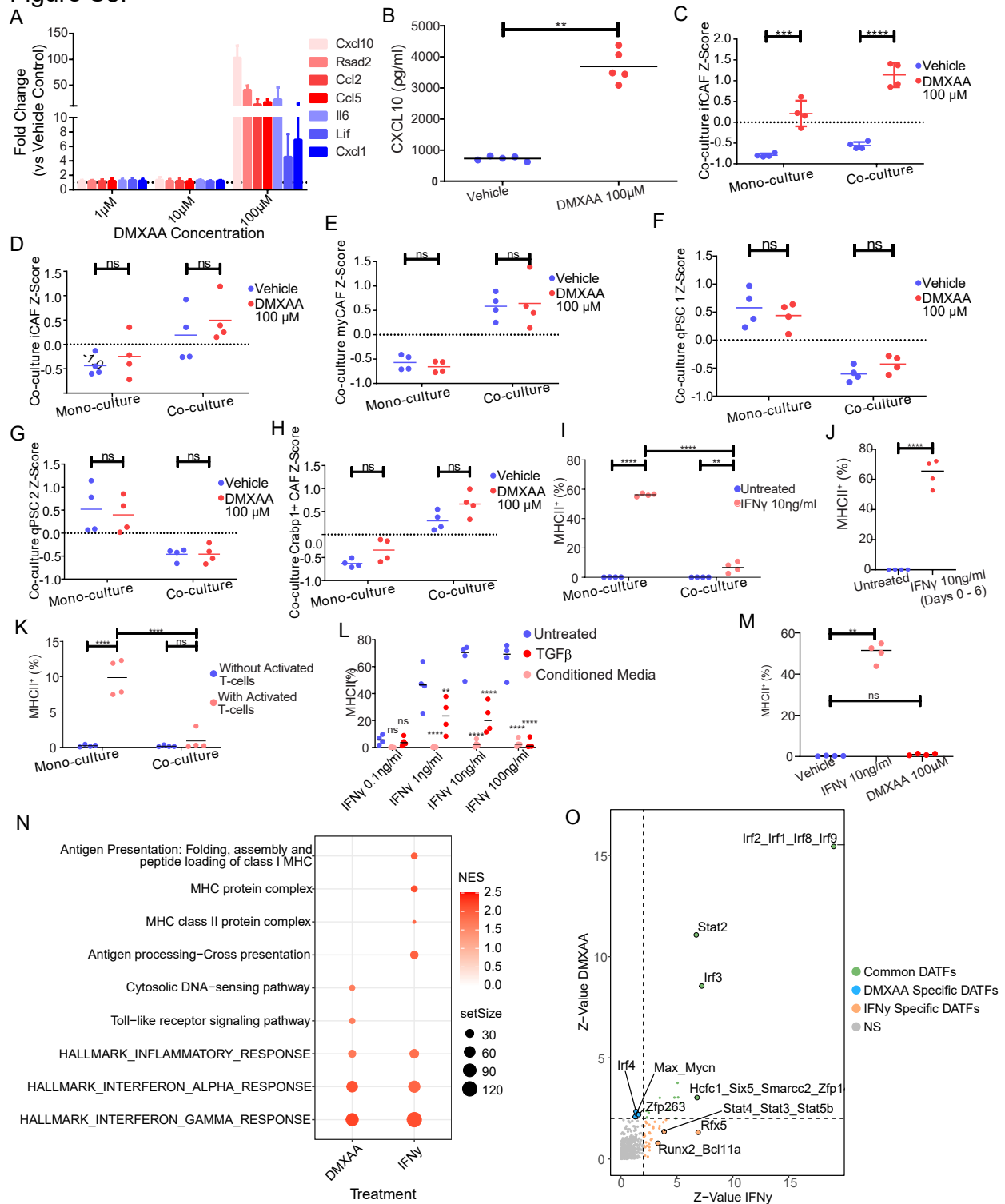

Supplement: Figure S8 — Type I and type II interferon pathways govern interferon-response CAF (ifCAF) and antigen-presenting CAF formation. [file can-23-3252_figure_s8_suppsf8.pdf]

Figure S9.

A

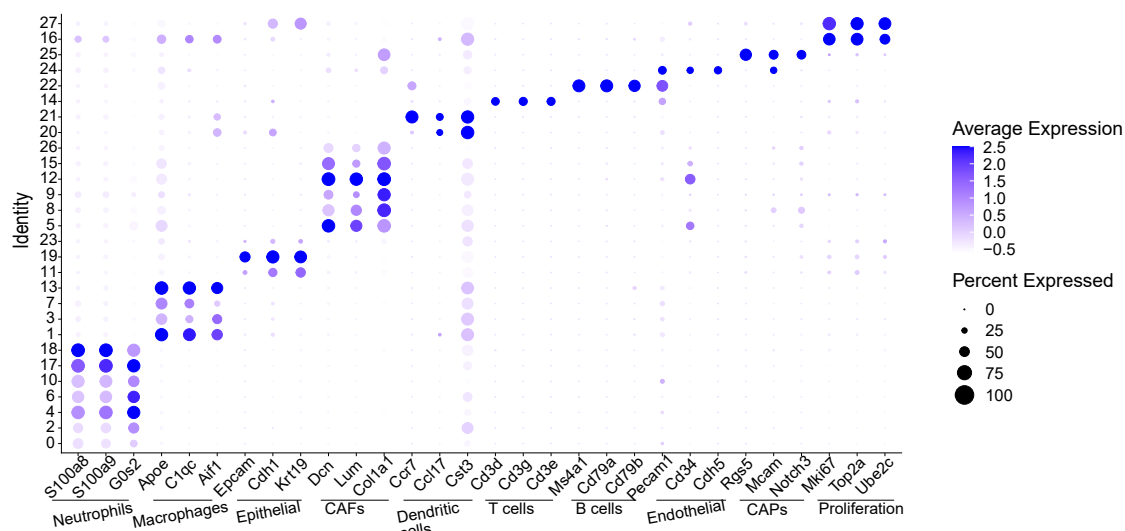

B

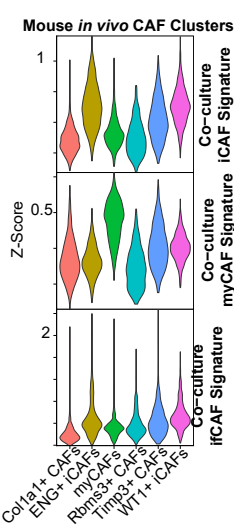

C

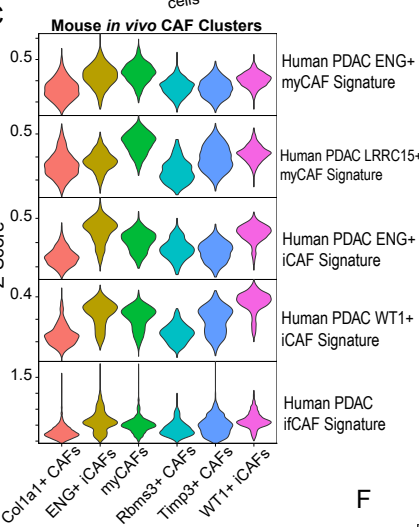

D

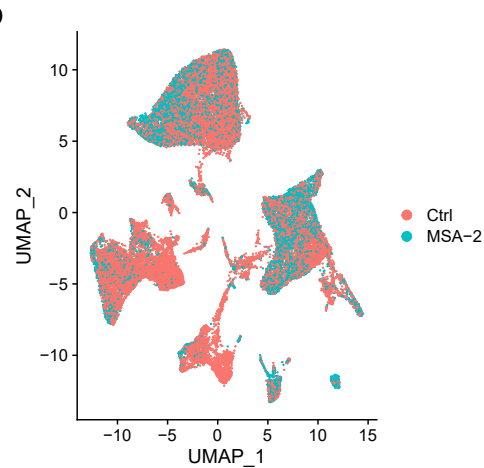

E

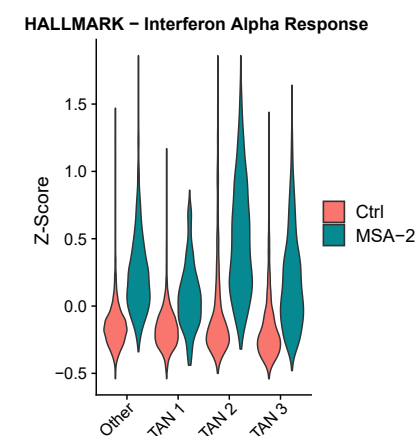

F

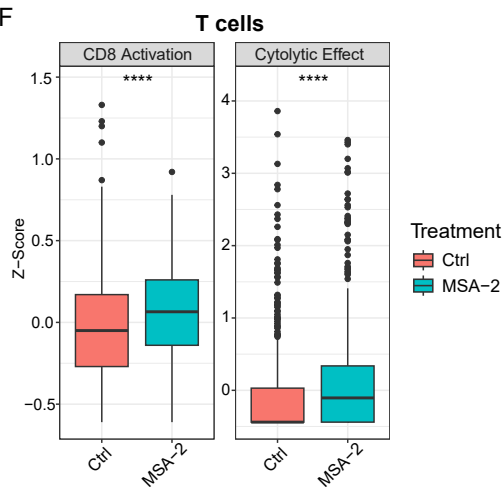

Supplement: Figure S9 — STING agonism reprograms the tumor microenvironment and suppresses metastasis in a mouse model of PDAC [file can-23-3252_figure_s9_suppsf9.pdf]

Figure S10.

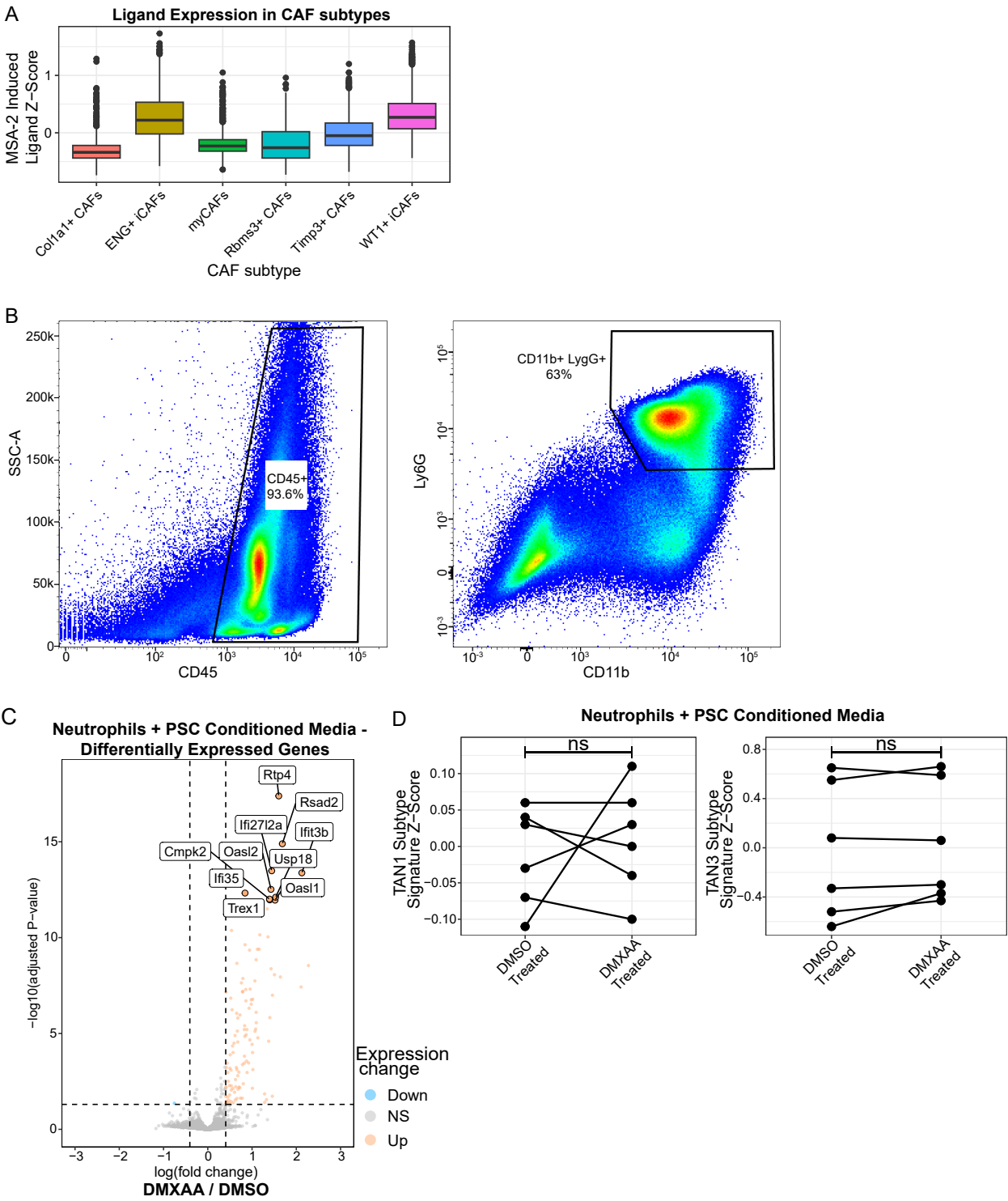

Supplement: Figure S10 — STING agonism in CAFs promotes TAN polarization. [file can-23-3252_figure_s10_suppsf10.pdf]

Figure S11.

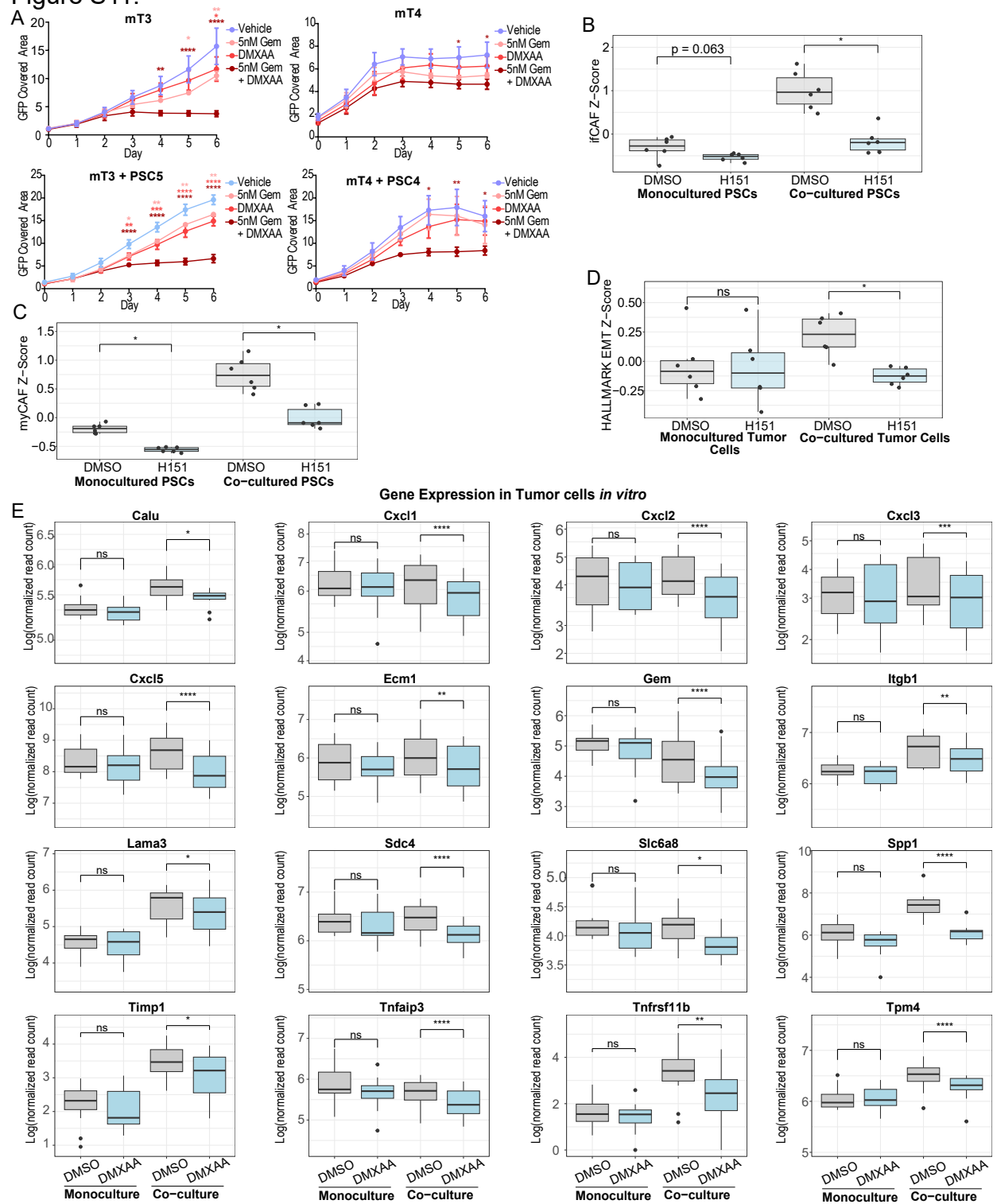

Supplement: Figure S11 — STING modulation in CAFs directs subtype formation which disrupts pro-metastatic influences of CAFs on tumor cells. [file can-23-3252_figure_s11_suppsf11.pdf]
